# Analytical and Chemometric Characterization of Fino and Amontillado Sherries during Aging in *Criaderas y Solera* System

**DOI:** 10.3390/molecules27020365

**Published:** 2022-01-07

**Authors:** Manuel J. Valcárcel-Muñoz, María Guerrero-Chanivet, María del Carmen Rodríguez-Dodero, María de Valme García-Moreno, Dominico A. Guillén-Sánchez

**Affiliations:** 1 Bodegas Fundador S.L.U. Departamento de Investigación y Desarrollo, C/San Ildefonso, n 3, 11403 Jerez de la Frontera, Cádiz, Spain; m.valcarcel@bodegasfundador.com (M.J.V.-M.); maria.guerreroch@uca.es (M.G.-C.); 2 Departamento de Química Analítica, Facultad de Ciencias, Instituto Investigación Vitivinícola y Agroalimentaria (IVAGRO), Campus Universitario de Puerto Real, Universidad de Cádiz, 11510 Puerto Real, Cádiz, Spain; maricarmen.dodero@uca.es (M.d.C.R.-D.); dominico.guillen@uca.es (D.A.G.-S.)

**Keywords:** fino, amontillado, sherry wine, criaderas y solera, wood, multiple linear regression

## Abstract

Fino and Amontillado are Sherry wines, produced in *Marco de Jerez* area (southern Spain), and aged in *Criaderas y Solera* system. Fino Sherry wine follows a biological aging process, under a veil of flor yeasts, while Amontillado Sherry wine shares the same biological aging firstly, followed by oxidative aging, which gives them special features. Organic acids, esters, higher alcohols, phenolic compounds and total dry extract of Sherries evolve during aging due to evaporation processes, physical-chemical reactions, wood contributions and microbiological activity. During aging, Sherry wines improve their organoleptic profile, as could be proved in the tasting sessions. Hierarchical Cluster Analysis and Factor Analysis with factor extraction using Principal Components of Sherry wines studied were carried out and natural groupings of the wines according to the type of aging and their age were observed. A strong correlation between the parameters analyzed and the aging of each wine has been seen in the Multiple Linear Regression studies, establishing two different models, one for each type of Sherry wine, that, with only four of all the variables studied estimated the wine age with more than 99% of confidence. This constitutes a useful tool to control the age of these Sherry wines in the winery.

## 1. Introduction

Sherry wines are one of the most appreciated wines in the world of oenology. Their uniqueness is due both to the grape variety that can be used for their production (Palomino, Moscatel and Pedro Ximénez), to the type of aging (biological, oxidative or mixed aging) and to the particular traditional dynamic system in which the wine is aged, known as *Criaderas y Solera*, as well as the climatic conditions of the only delimited area in which their production can be carried out: the *Marco de Jerez* (Protected Designation of Origin).

The Fino wine is described as pale in color, very dry, with light acidity and sharp aromas with hints of almonds. This Sherry wine is made from the Palomino grape variety, aged with the constant presence of a film of indigenous yeasts on the surface of the wine, called the veil of flor yeasts, giving rise to the aging process known as Biological Aging. The development of these yeasts begins on the surface of the wine at the end of the alcoholic fermentation of the must, developing an aerobic metabolism that consumes, and at the same time produces, a series of compounds that provide particular organoleptic characteristics [1]. The alcoholic strength during the aging process of this wine is between 15.0 and 15.5% vol. and it must not be exceeded in order to guarantee the development of the flor yeasts that participate in its aging. The Amontillado wine, which is also made from the same Palomino grape, is first aged in a Biological Aging process of Fino wines at 15.0–15.5% vol., and after a subsequent fortification to a minimum of 16.0% vol., which avoids the feasibility of the development of that yeast film on the surface of the wine, is aged by means of a traditional oxidative wine system, the Oxidative Aging, which is the reason why the aging of this wine is called mixed aging. Under these aging conditions, the Amontillado wine is characterized by acquiring an amber tone during aging and by having very unique organoleptic properties, as it combines the memory of the biological aging with the woody notes acquired during its oxidative stage [2,3].

Sherry wines are aged in traditional dynamic systems of *Criaderas y Solera*. These systems consist of oak casks, generally American oak (*Quercus alba*), with a capacity of 500–600 L, grouped according to the age of the wine on various levels and/or scales. Each aging level is known as *Criadera*, except for the last level, the oldest one, known as *Solera*, which is where the Sherry wine for consumption is supplied. In this dynamic system, periodic movements are carried out, where a part of the Sherry wine contained in each of the oak casks forming the same aging level is extracted and replaced with Sherry wine extracted from the next aging level [4,5,6]. The *Criaderas y Solera* system facilitates the maintenance of the physicochemical and organoleptic characteristics of Sherry wine of the Solera level, removing any possible heterogeneities that could arise in the characteristics of the young fortified wines that are used each year to replenish the system.

Casks are an essential element in the aging process of wines. They are said to be active containers since, during aging, they release numerous compounds into the wine and participate in chemical reactions with them, exerting a great influence on them and modifying their organoleptic properties, improving them considerably [7,8,9]. Wood, in particular, provides phenolic compounds including phenolic aldehydes [10] derived from benzoic acid (such as vanillin and syringaldehyde) and from cinnamic acid (such as conieraldehyde and sinapaldehyde), which are derived from lignin, which is thermally degraded during the barrel-making process [11,12]. Among the compounds yielded by wood, furfurals and their derivatives from wood hemicellulose are worth mentioning [11]. In addition to phenolic compounds, wood also provides other substances, such as fatty acids, inorganic substances and alcohols, among others, which enrich the wine during aging and increase its quality.

In this work, a complete physicochemical characterization of a *soleraje* of Fino wine and another one of Amontillado wine, related to each other, has been carried out, highlighting the differences between Biological Aging and Oxidative Aging, which are subsequently reflected in the tasting sessions of these wines. In addition, a chemometric study was carried out using unsupervised pattern recognition techniques, namely Cluster Analysis and Factor Analysis with factor extraction using Principal Components. As a result, natural clusters of samples have been observed in two clearly differentiated groups, depending on whether they have been aged under biological aging or in an oxidative environment. Likewise, Multiple Linear Regression (MLR) studies were carried out, where strong correlations were observed between the parameters analyzed and the aging of the wine, allowing the establishment of two models, one for each type of wine, with only four variables each, being able to estimate the age of Fino and Amontillado wines with more than 99% accuracy.

Chemometrics is a powerful chemical tool for establishing relationships and models from a matrix of analytical data [13,14,15]. In this case, it has enabled us to identify the most influential parameters in the aging of the two types of wines studied in this work, turning into a very useful tool for the industry and for routine cellar analysis, since by determining only four parameters, it has been possible to estimate the age of both Fino and Amontillado wines in an easy and quick way.

## 2. Materials and Methods

### 2.1. Samples

**Figure 1:**
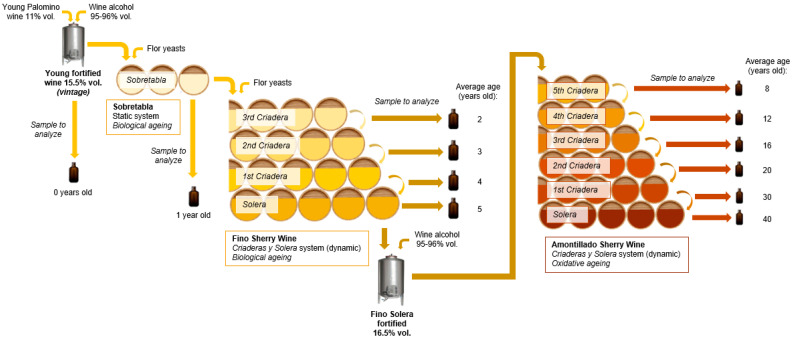
Diagram of the studied aging process and sampling.

The sherry wines, the wine distillates used for the fortifications and the casks used in the study were supplied by Bodegas Fundador, S.L.U. (Jerez de la Frontera, Cádiz, Spain), as well as the aging cellar facilities where this study was carried out. The casks selected for the study, belonging to the industrial *solerajes* of Bodegas Fundador, S.L.U., were made of *Quercus alba*, medium toast and 500/600 L capacity. All of them were supplied by cooperages located in Jerez de la Frontera, Spain. The studied process diagram is shown in Figure 1.

In the static aging stage of the Sobretabla, 100 casks of 600 L were selected. Similarly, 100 casks of 500 L were selected in each scale from the Fino *soleraje* (composed of three Criaderas and one Solera). For the Amontillado *soleraje*, 50 casks of 500 L were chosen for the 2nd, 3rd, 4th and 5th Criadera qualities and 25 casks of 500 L for the 1st Criadera and Solera qualities (given that, due to their high aging, the aging stock was less than 50 casks).

During the four years of the study, the young wine of the year (grape variety: Palomino) at 11% vol. was fortified with distilled wine alcohol (95–96% vol.) until it reached an alcohol content of 15.5% vol. (vintage). Each year, in November, once the grape harvest is finished, the necessary volume of young fortified wine (vintage) is selected to fill 100 casks of 600 L capacity with 540 L of wine for the future Sobretabla quality and to age for a year under the presence of a veil of flor yeasts on its surface, leaving a vacuum volume to allow the filmogenic development.

The aged vintage wine (average age of one year), called *Sobretabla*, marks the beginning of the *soleraje* of Fino wine (Figure 1). The aging of the Fino wine, as indicated in its specifications, was carried out under the presence of a veil of flor yeasts on its surface (Biological Aging). In the system studied, the average accumulated age of the wine in the 3rd Criadera is 2 years old; in the 2nd Criadera, 3 years old; in the 1st Criadera, 4 years old; and in the Solera, 5 years old. The condition of the veil of flor yeasts was periodically checked to ensure its presence, and on those occasions when some casks showed a loss of vigor, they were replanted with flor yeasts from the veil of the casks adjacent to the affected ones.

The aging of the Amontillado began after the Solera of the Fino wine was removed. For this purpose, the wine extracted from the Solera of Fino wine was fortified with distilled wine alcohol (95–96% vol.) until it reached an alcohol content of 16.5% vol. so that no flor yeasts could develop. From that point, the wine entered Oxidative Aging. Figure 1 shows the constitutional scheme of the *soleraje* of *Criaderas y Solera* of Amontillado, where the average accumulated age of the wine in the 5th Criadera is 8 years old; in the 4th Criadera, 12 years old; in the 3rd Criadera, 16 years old; in the 2nd Criadera, 20 years old; in the 1st Criadera, 30 years old; and in the Solera, 40 years old.

Samples of the wine contained in the casks were taken in May every year during the four years of the study. In these samplings, equal volumes were taken from each of the casks belonging to the same quality, whether Sobretabla, Fino, or Amontillado, obtaining samples of 5 L for each quality, representative of the total number of casks that made up each of the aging scales and type of wine studied; samples were also taken from the young wine fortified at 15.5% vol. (vintage-aging: 0 years old), in November of each year (Figure 1). All these samples together (four samples for each type of wine and aging, n = 4) were physicochemically and sensorially characterized each year. All analyses were carried out in duplicate.

### 2.2. Reagents

For the preparation of the eluent for the determination of citric, tartaric, malic, succinic and lactic acids, ultrapure deionized water (EMD Millipore, Bedford, MA, USA); 0.2N concentrated sulphuric acid (Sigma-Aldrich, Saint Louis, MO, USA) and UHPLC grade acetone (VWR International, Radnor, PA, USA) were used.

For the determination of the Folin-Ciocalteau Index, ultrapure deionized water (EMD Millipore, Bedford, MA, USA), Folin-Ciocalteau reagent (Merck, Darmstadt, Germany) and anhydrous sodium carbonate (Merck, Darmstadt, Germany) were used. Gallic acid (Merck, Darmstadt, Germany) was used as a calibration standard. 

UHPLC-grade acetonitrile (Panreac, Barcelona, Spain), acetic acid (Merck, Darmstadt, Germany) and ultrapure deionized water (EMD Millipore, Bedford, MA, USA) were used in the preparation of the UHPLC phases for the determination of phenolic and furfural compounds.

Standards for calibrations and reagents for routine oenological parameter analyses were supplied by Sigma-Aldrich (Saint Louis, MO, USA).

### 2.3. Oenological Control Parameters

All the oenological control parameters were carried out according to the official methodology described by the OIV in the Compendium of International Methods of Analysis of Wines and Musts. The alcoholic strength (% vol.) was determined by distillation of the wine and subsequent measurement of the density of the distillate [16] with a DMA-5000 digital density meter (Anton Paar, Ashland, OR, USA). The density of the samples was also measured directly on a DMA-5000 digital density meter (Anton Paar, Ashland, OR, USA) and was expressed in g/L. The pH was determined using a Basic 20 pH meter (Crison Instruments SA, Barcelona, Spain). Total acidity was determined by potentiometric titrations at pH 7 [17] and was expressed in g tartaric acid/L. Volatile acidity was determined using an AA3 HR Autoanalyzer segmented flow analyzer (Seal Analytical, Norderstedt Stadt, Germany) according to the official method [18] and the results were expressed in g acetic acid/L. The glycerol content in mg/L was determined according to the official enzymatic method [19]. The total sulfur dioxide in mg/L was determined according to the Ripper method [20]. The amount of sulfates, expressed in g potassium sulfate/L, was determined by the gravimetric method of barium sulfate precipitation [21], while phosphates, expressed in mg phosphate/L, were determined by the colorimetric method of vanadomolybdophosphoric acid [22]. The concentration of potassium and calcium, in mg/L, was obtained by atomic absorption spectroscopy with a PinAAcle 900F (Perkin Elmer, Boston, MA, USA) equipped with WinLab32 AA software (Perkin Elmer, Boston, MA, USA). Total dry extract, expressed in g/L, was determined by gravimetry [23]. Reducing substances, expressed in g/L, were also obtained according to the official procedure [24]. All analyses were carried out in duplicate. Sugar-free extract, expressed in g/L, was obtained through the following formula [23]:

$$Sugar-free\;extract\;\left(g/L\right)=Total\;dry\;extract\;\left(g/L\right)-Reducing\;substances\;\left(g/L\right)$$

### 2.4. Organic Acids

Citric, tartaric, malic, succinic and lactic acids were determined by ion chromatography with a 930 Compact IC Flex (Metrohm, Madrid, Spain), equipped with a Metrosep Organic Acids column of 250 mm × 7.8 mm (i.d.) and 9 µm particle size. A mixture of deionized water:acetone:sulfuric acid (84:12:4), at a flow rate of 0.4 mL/min, was used as eluent. The software used for data acquisition and processing was MagicNet 3.3 (Metrohm, Madrid, Spain). Compounds were identified by a comparison of the retention time and the standard used. Results are expressed in mg/L. The standards and samples were injected in duplicate. 

### 2.5. Acetaldehyde, Acetal, Acetoin, Methanol, Esters and Higher Alcohols

Acetaldehyde, acetaldehyde-diethylacetal, acetoin, methanol, esters (ethyl acetate, ethyl lactate, ethyl succinate, ethyl caproate, ethyl caprylate, ethyl caprate, ethyl laureate, ethyl myristate and ethyl palmitate) and higher alcohols (n-propanol, isobutanol, 2-methyl-1-butanol, 3-methyl-1-butanol, hexanol and 2-Phenyl ethanol) were determined by GC-FID. These analyses were performed on an Agilent 7890B Gas Chromatograph (Agilent Technologies, Santa Clara, CA, USA) coupled to a flame ionization detector. A DB-624 column (30 m × 250 µm × 1.4 µm, Agilent Technologies, Santa Clara, CA, USA) was used for the analysis of acetaldehyde, acetaldehyde-diethylacetal, methanol, ethyl acetate and higher alcohols. The column used for the analysis of acetoin and esters (except ethyl acetate) was the CP-WAX 57 CB (25 m × 250 µm × 0.2 µm, Agilent Technologies, Santa Clara, CA, USA). The methodology used is described in previous works of our research group [25]. Samples were injected directly and in duplicate. Results were expressed in mg/L. 

### 2.6. Folin-Ciocalteau Index

The total content of phenolic compounds was determined using the Folin-Ciocalteau Index (FCI) [26]: 1 mL of sample, 50 mL of ultrapure deionized water, 5 mL of Folin-Ciocalteau reagent and 20 mL of 20% anhydrous sodium carbonate solution were added to a 100 mL flask and ultrapure deionized water was added up to the mark in this order. After shaking the flask and waiting 30 min, Absorbance was measured at 750 nm in a Lambda 25 spectrophotometer (Perkin Elmer, Boston, MA, USA), using 10 mm light path glass cuvettes. The results obtained in the analyzed samples were expressed as mg gallic acid/L. Previously, a calibration curve was performed with gallic acid, in a concentration range between 0 mg/L and 750 mg/L, obtaining R^2^ = 0.9998. The standards and samples were injected in duplicate.

### 2.7. Phenolic Compounds and Furfurals

Phenolic and furfural compounds were quantified by UHPLC following the methodology developed by our research group [25,27]. The equipment used was a Waters Acquity UPLC equipped with a PDA detector and an Acquity UPLC C18 BEH column, 100 mm × 2.1 mm (i.d.) and 1.7 µm particle size (Waters Corporation, Milford, MA, USA). Fifteen phenolic compounds were identified (gallic acid, p-hydroxybenzoic acid, vanillic acid, syringic acid, protocatechuic acid, caffeic acid, trans-caftaric acid, p-coumaric acid, cis-coutaric acid, trans-coutaric acid, ferulic acid, fertaric acid, p-hydroxybenzaldehyde, vanillin and syringaldehyde) and two furanic aldehydes (5-hydroxymethylfurfural and furfural). Samples and standards were filtered through nylon membranes with a pore size of 0.22 µm and injected in duplicate. The analyzed compounds were identified by comparison of the retention time and the UV-Vis spectrum of the sample and by the standard used. Results were expressed in mg/L.

### 2.8. Color Measurements

The color of the samples was determined by measuring Absorbance in a Lambda 25 spectrophotometer (Perkin Elmer, Boston, MA, USA), according to the official method established by the International Organisation of Vine and Wine (OIV) [28]. The wavelength studied in these samples was 470 nm, related to the yellow-golden-amber shades, given their relevance in Fino and Amontillado wines. All results were expressed in Absorbance units, and the measurements were made in duplicate.

### 2.9. Tasting Sessions

The tastings were carried out in a room whose characteristics facilitated the concentration and isolation of the tasters, who worked individually and at a room temperature of 20 °C [29]. The study involved seven tasters, all members of the company’s tasting panel and with more than 10 years of experience in sherry wine tasting. Additionally, four of them are members of the official tasting panel of the Designation of Origin Jerez, Xérès, Sherry.

From among the numerous samples that have been studied from a physicochemical point of view, two Amontillado wines of very different average ages (12 years old and over 30 years old) were selected for the tasting, together with the Fino wine of 5 years old of average age and the fortified wine of the year (vintage) that was used in the last replenishment of the Sobretabla.

Fifty mL of each sample were poured into black glass wine [30] containers and covered with a glass lid to stabilize the headspace for at least ten minutes prior to tasting. Nose and mouth perception data were taken. The four samples of the study were evaluated in duplicate.

The descriptors were selected according to the tasting criteria used in the Designation of Origin Jerez, Xérès, Sherry. Table 1 lists the definitions of these descriptors, as well as the olfactory-taste patterns for the maximum intensity (value: 9) of each, on the 9-point interval numerical scale used [31]. A young wine of the year of the Palomino variety (11% vol.) was used as the minimum intensity standard (value: 1).

### 2.10. Statistical Analysis

Statgraphics 19 software package (Statgraphics Technologies, Inc., The Plains, VA, USA) was employed for ANOVA, Fisher’s Least Significant Difference test, Hierarchical Cluster Analysis, Factorial Analysis with factor extraction using Principal Component and Multiple Linear Regression analysis. Other statistical parameters were performed in Microsoft Excel 2016 (Microsoft Corp., Redmond, WA, USA).

The statistical treatment of the tasting sessions included an analysis of variance taking the sample as a factor of variation and a Factor Analysis. Statistica 8.0 software was used for both. (StatSoft Inc., Tulsa, OK, USA). The spider chart was made in Microsoft Excel 2016 (Microsoft Corp., Redmond, WA, USA).

## 3. Results and Discussion

### 3.1. Oenological Control Parameters of Sobretabla, Fino and Amontillado Sherry Wines

#### 3.1.1. Alcoholic Strength

Two very different trends can be observed depending on the type of aging of the studied wines (Table 2). In the case of the Sobretabla and the Fino wines, both with biological aging, there is a slight decrease in alcoholic content with respect to the young fortified wine (vintage), and then it remains more or less constant in the older wines. The flor yeasts present in the biological aging of Sobretabla and Fino consume part of the alcohol present in the medium and use it for their cellular regeneration as a source of carbon [32]. For this reason, small alcoholic adjustments with distilled wine alcohol were made to the replenishment wine, each time extraction and replenishment operations were carried out in the different stages of aging, in order to guarantee an alcohol content between 15 and 15.3% vol. in the wine inside the casks. This explains why the alcoholic strength determined is more or less constant and has not decreased as much as if these corrections had not been made.

In the case of the Amontillado wines, which are aged in an oxidative environment, an increase in alcohol content is observed as the wines get older. This is mainly due to the transpiration of water molecules through the pores of the wood, which generates a reduction in volume, traditionally known as *merma* or volume reduction [33], and which causes the concentration of other compounds present in the medium, such as alcohol in this case. Table 3 shows, for each average aging period studied and on the basis of a *merma* of 3% per year (average value obtained during the 4 years of this study), the theoretical increase in concentration that the rest of the non-volatilizable compounds would undergo. In the case of the Fino wines, the annual decrease in volume was as much as 4.5%, as a consequence of the biological activity. Obviously, the casks do not reach these stock volumes, as each time the volume of wine is replenished with the next oldest wine, partly compensating for this effect.

#### 3.1.2. pH, Total Acidity and Volatile Acidity

A decrease in pH and total acidity (Table 2) was observed in those wines aged under biological aging (Sobretabla and Fino wines). This decrease may be due, in part, to the precipitation of salts, such as potassium bitartrate, calcium tartrate and calcium sulfate. In addition, the flor yeasts and/or bacteria involved in the biological aging of Sobretabla and Fino wines can metabolize or produce part of the organic acids present in the wine [34], justifying this decrease, which also increases with the age of the wine, with the Fino wine of the Solera having the lowest pH and total acidity values. The trend observed in the volatile acidity for these wines is also downward, as a consequence of the metabolism of the flor yeasts. Again, the lowest values are observed in the Fino wine of the Solera. During the first stages of biological aging of Sobretabla and 3rd Criadera of Fino, in addition to the biological activity of the flor yeasts, there is a slight malolactic fermentation carried out by the lactic bacteria [35] and even these same bacteria metabolize the existing gluconic acid [36]. This causes the generation of acetic acid [37] and, as a consequence, an increase in the volatile acidity, although sometimes this increase can be counteracted, in part, by the flor yeasts, which metabolize the acetic acid as it is produced.

In the case of the Amontillado wines, a completely opposite evolution is observed: there is an upward trend as the aging time passes. The oxidation and esterification processes, the transfer of acids from the wood and the *merma* (Table 3) justify the increase in total acidity and volatile acidity as these Amontillado wines get older. In the case of pH, there is also an increase and this is partly due to the oversaturation of potassium in the wine. Although precipitation of compounds derived from organic acids, such as tartaric acid, is also observed, in this case, it is compensated by the already mentioned phenomena.

Given the aforementioned precipitations and the oversaturation of potassium, it is very important to carry out cold stabilization processes before these wines are put on the market [38].

#### 3.1.3. Total Sulfur Dioxide 

In the case of total sulfur dioxide (Table 2), a decrease in concentration is observed as aging time increases, regardless of whether they are Sobretabla, Fino, or Amontillado wines. This compound oxidizes to sulfate and is, therefore, depleted as the wine ages (it transforms). 

#### 3.1.4. Sulfates and Phosphates 

The sulfate content (Table 2) shows a decreasing trend in both Sobretabla and Fino wines and an increasing trend in the case of the Amontillado wines. The sulfates present in the wine are derived both from the grapes and from the plastering, a traditional practice carried out during the harvest in this warm climate area of Jerez in order to reduce the dose of tartaric acid in the acidity correction and improve the fermentation and the aromatic characteristics of the young wine [39]. In the first stages of the aging process studied, Sobretabla and Fino wines (1–5 years old), a decrease in concentration is observed, which can be attributed to the precipitation of calcium sulfate. In the Amontillado wines, where the aging of the wine exceeds 5 years, its increase could be justified by the *merma*, although these contents, lower than expected, show that there must still be precipitation of calcium sulfate.

In the Fino and Sobretabla wines, the phosphate content (Table 2) decreases, because they can be a source of phosphorus in the cell development of the flower yeasts [34]. It can also precipitate as calcium phosphate, which is also the reason for the decrease in calcium phosphate. As with sulfates, an upward trend is observed in Amontillado, which can be attributed to the *merma* effect.

#### 3.1.5. Potassium and Calcium

Regarding potassium (Table 2), there is a significant decrease in its concentration compared to the young fortified wine (vintage) in the Sobretabla, as well as a further significant decrease in the 3rd Criadera of Fino. This decline then continues slightly, and, from the 1st Criadera of Fino, there is an increase in potassium with aging. Potassium, together with tartaric acid, gives rise to potassium bitartrate, whose solubility in wine depends on the alcoholic strength. Wine is usually supersaturated with potassium bitartrate, so when the temperature inside the cellar is low (10–12 °C), the precipitation of potassium bitartrate is accentuated inside the casks. This explains the decreases in potassium observed during the first stages of aging. From the Solera of Fino onwards, this effect can be counteracted by the increase in concentration due to *merma* (Table 3), in addition to the fact that the wood is capable of releasing it, albeit in very small quantities [40], and therefore, its effect is not significant. In the older Amontillado wines, potassium is very oversaturated and is partly responsible for the increase in pH.

The behavior of calcium (Table 2) is similar to that of potassium, except that, in this case, the precipitations produced are calcium tartrate and calcium sulfate salts, which are very insoluble in alcohol.

#### 3.1.6. Glycerol

Glycerol is present in wine as a by-product of alcoholic fermentation [34]. A decrease of this compound is observed in both Sobretabla and Fino and an increase in Amontillado (Table 2). In biologically aged wines, flor yeasts use it as a source of carbon in their metabolism, which justifies its decrease (more than 90% in the Solera of Fino). In the case of Amontillado wines, whose aging is oxidative, what is observed is a concentration effect due to the *merma*.

#### 3.1.7. Density, Total Dry Extract, Reducing Substances, Sugar-Free Extract

In Sobretabla and Fino wines there is a decrease in density and total dry extract compared to the young fortified wine (vintage) (Table 2). Moreover, the decreasing trend is maintained as the aging time goes by. This may be due, in addition to the precipitation already mentioned in previous sections, to the fact that flor yeasts use glycerol as a carbon source for their development [34], which affects the total dry extract of the wine. The concentration of glycerol drops sharply with biological aging and makes Fino the wine with the lowest total dry extract in the world [41], generating organoleptic sensations of dryness in the mouth, as could be proved in the tasting sessions (Section 3.9). 

In the case of the Amontillado wines, the total dry extract increases as a consequence of the concentration derived from the *merma* of all those compounds that define it (acids, glycerol, polyphenols, reducing substances-pentoses, hexoses and polysaccharides extracted from the wood, etc.). 

Reducing substances present in wine are mainly composed of sugars and polysaccharides. In the young fortified wine (vintage) there may remain traces of sugars, mostly fructose, from the alcoholic fermentation of the young Palomino wine of the year 11% vol. During the biological aging process in the Sobretabla, the flor yeasts use these residual sugars (1–2 g/L) as a source of carbon in their metabolism [37], which generates a decrease in the concentration of reducing substances in the wine, whose decreasing tendency is maintained in the Fino wines as they age. In the case of Amontillado wines, which are aged under oxidative aging, a change of trend is observed, which now becomes ascending over time. This may be due to the transfer from the wood of substances, such as pentoses, hexoses and/or polysaccharides from the hemicellulose [42,43]. The longer the aging time, the greater the transfer and, consequently, the greater the reducing substances present in the oldest Amontillado wines.

The sugar-free extract is a measure of all those non-volatile substances present throughout the years of aging in the wine, without taking into account those of a reducing nature (sugars, polysaccharides, etc.). In the Sobretabla and Fino wines, a decrease is observed, mainly due to the impact of the biological activity of the flor yeasts. However, in Amontillado, there is a significant increase with age, which may be due to contributions from the wood [44] and the concentration of compounds derived from the *merma*.

### 3.2. Organic Acids Present in Sobretabla, Fino and Amontillado Sherry Wines

The origin of the organic acids present in the wines can be very diverse: the grape itself, alcoholic fermentation and/or malolactic fermentation. Table 4 shows that in the earliest stages of biological aging (Sobretabla and Fino), there is a significant decrease in malic acid, which comes from the grapes, as it is consumed by lactic bacteria and the flor yeasts. On the other hand, in these stages, an increase in lactic acid concentration is observed (Table 4), as it is produced by lactic bacteria during malolactic fermentation. In the older Fino scales, malic acid follows a decreasing trend, while lactic acid changes its increasing trend to decreasing, as it is metabolized by the flor yeasts [34]. Citric acid (Table 4), which is part of the metabolic pathway of the yeasts during the development of the veil of flor yeasts [34,45], evolves in a similar way to malic acid, as it decreases with aging time in biologically aged wines. Succinic acid, generated during alcoholic fermentation [34], behaves in the same way. In the case of Amontillado wines, these four acids evolve in the same way: their concentration increases due to the *merma* effect (Table 3).

The tartaric acid (Table 4) present in the wine comes both from the grapes and from the addition of tartaric acid at harvest to adjust the pH in areas with warm climates. Its evolution is different from the rest: it has a decreasing trend with aging time in all the wines studied (Sobretabla, Fino and Amontillado), as a consequence of the precipitations of potassium bitartrate and calcium tartrate that take place during the aging process.

### 3.3. Acetaldehyde, Acetal, Acetoin, Methanol, Esters and Higher Alcohols in Sobretabla, Fino and Amontillado Sherry Wines

Table 5 shows the amounts of acetaldehyde, acetaldehyde-diethylacetal, acetoin, ethyl acetate, methanol, higher alcohols, ethyl esters of organic acids and ethyl esters of fatty acids in the Sobretabla, Fino and Amontillado wines studied. Acetaldehyde and acetaldehyde-diethylacetal, both related to the metabolism of flor yeasts [46], follow an increasing trend in those wines whose aging has been biological (Sobretabla and Fino), reaching its maximum value in the Fino of the Solera, the oldest aging scale within this type of aging. The species *Sacch. cerevisiae*, *race montuliensis* produces more acetaldehyde than *Sacch. cerevisiae*, *race beticus*, justifying the increase of acetaldehyde with aging [47] as the former is more present in the older scales (Table 6); in the case of Amontillado wines, it is observed that the values of both compounds decrease, due to oxidation to acetic acid and subsequent esterification to ethyl acetate.

The concentration of ethyl acetate increases in the Sobretabla with respect to the young fortified wine (vintage) due to the malolactic fermentation that takes place in the initial stage and is related to bacterial activity [34]. In the Fino wines, this compound decreases as the flor yeasts metabolize it [37,45,49], reaching its lowest concentration in the Fino of the Solera. In Amontillado wines aged under oxidative aging, a considerable increase of this compound is observed, due to the oxidative state in which it ages, which favors oxidation to acetic acid and subsequent esterification with alcohol. In addition to this effect, there is the effect of the *merma*, although it is comparatively insignificant. 

In biological aging, flor yeasts are responsible for the increase in the concentration of acetoin in the wine, given that in the metabolic pathway followed by the yeasts [47], they transform two molecules of acetaldehyde into one of acetoin (the greater presence of acetaldehyde facilitates the increase). In the case of Amontillado, the value of this compound remains more or less constant, without a clear trend, reflecting that there is a degradation similar to the effect of the *merma*.

Regarding methanol, no clear trend is observed in the Sobretabla and in the younger Fino wines, however, in the older Fino wines and in all the Amontillado an increase is observed with aging time, probably due to the *merma*.

The values of n-propanol, isobutanol, 2-methyl-1-butanol and 3-methyl-1-butanol increase with aging time in all the wines studied, either by metabolic routes by the flor yeasts from amino acids [34], in the case of the wines under biological aging, or by the effect of *merma* in those under oxidative aging (Table 3). 2-phenylethanol is produced by yeasts during alcoholic fermentation and shows an increasing evolution with respect to the aging time in all the wines studied, which is attributed to the concentration by the *merma*.

Esters derived from organic acids (ethyl lactate and diethyl succinate) increase their concentration in all the wines studied with aging, especially in Amontillado, where the presence of their acids in significant quantities facilitates esterification with the alcohol.

The evolution of fatty acid ethyl esters is very plane, except in the case of ethyl caprylate, which, although it follows an upward trend in the Sobretabla and in the Fino, shows a very notable increase in the case of Amontillado. This may be related to a residual biological activity, in addition to the corresponding increase in the *merma*. By fortifying the Solera of Fino from 15% vol. to 16.5% vol. to replenish the last criadera of Amontillado, lysis of the residual yeast cells can occur and release these substances into the wine, as both these esters and their acids are present in the flor yeasts [37]. 

### 3.4. Phenolic Composition and Folin-Ciocalteau Index of Sobretabla, Fino and Amontillado Sherry Wines

All the wines studied showed an increase in Folin-Ciocalteau Index (FCI) with aging time (Table 7). This is due to the contribution of phenolic compounds released by the wood in the barrels [11,12]. Moreover, this increase is more marked in oxidative environments (oxidative aging). 

The same trend is observed for the phenolic and furfural compounds analyzed. All 15 compounds studied were found in young wines fortified before aging (Table 7). Gallic acid, caffeic acid and tartaric acid derivatives (trans-caftaric, cis-coutaric, trans-coutaric and fertaric acids) are found in higher concentrations. 

In general, the concentration of compounds transferred from wood, such as gallic, p-hydroxybenzoic, vanillic and syringic acids; p-hydroxybenzaldehyde, vanillin and furfurals increase with aging, as a consequence of the increased transfer. It is worth noting the strong increase observed for 5-hydroxymethylfurfural and furfural in very aged wines (older Amontillado wines). 

Those phenolic compounds, such as those derived from tartaric acid, p-coumaric acid, or ferulic acid, whose origin is exclusive to grapes, are hydrolyzed, generating an increase in their acids. Likewise, other compounds, such as trans-caftaric acid, which decreases in concentration during aging due to hydrolytic reactions, also generate a release of caffeic acid in the wine (Table 7).

### 3.5. Evolution of Color during Aging of Sobretabla, Fino and Amontillado Sherry Wines

To study the evolution of color in the wines, absorbance at 470 nm was determined, as this wavelength reflects the yellow - golden - amber tones acquired by the wines as they age. Both wines aged under biological aging (Sobretabla and Fino) and those aged under oxidative aging (Amontillado) show an increase in the absorbance value with aging time (Table 8), as expected. This may be due to oxidations of compounds already present and/or transfers of compounds from the wood [11,12,25], which is more marked in the oxidative environment where Amontillado wines age. The increase in color is lower in the Sobretabla and the Fino wines, as the veil of flor yeasts maintains a reducing medium that prevents polyphenols from oxidizing [50,51].

### 3.6. Hierarchical Cluster Analysis of Fino and Amontillado Sherry Wines

In order to study the natural groupings of the wines studied in this work, a Cluster Analysis was carried out, taking into account all the physicochemical variables studied in the work except those that, in one way or another, can be altered and/or corrected by the addition of compounds during oenological practice (such as alcohol content, pH, total sulfur dioxide and methanol). Ward’s clustering method and Euclidean distance metric were followed. The clustering was performed on the basis of the observations (vintage, Sobretabla and Sherry wines), which totaled 48.

**Figure 2:**
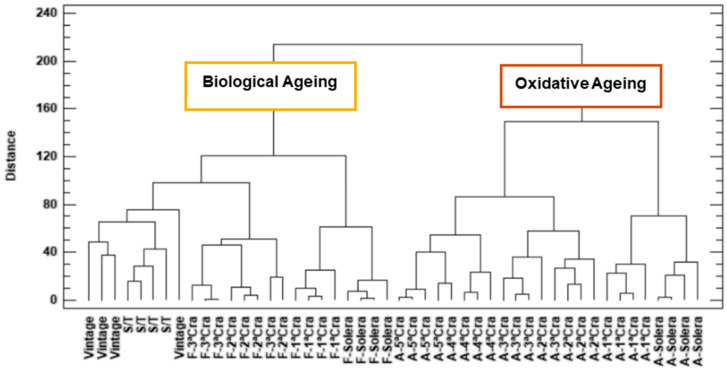
Cluster analysis (Ward’s method, Euclidean distance) of the Sherry wines studied.

In the obtained dendrogram (Figure 2), two main groups were observed. All the cases studied are ordered from left to right according to the age of the wine. The first cluster that can be observed corresponds to the group of younger wines (vintage, Sobretabla and Fino wines) and which, in turn, have been aged under biological aging. The second cluster observed is the group of Amontillado wines, ordered, from left to right, also by age. This would be the group of samples that aged in an oxidative environment (after biological aging).

### 3.7. Factor Analysis with Factor Extraction Using Principal Component

A Factor Analysis was carried out with extraction of factors by means of Principal Components in which, starting from 51 different variables, five factors were obtained that explain 93.7% of the total variability of the data obtained. 

In order to perform this analysis, all the physicochemical variables studied in the work were taken into account, except those that, in one way or another, can be altered and/or corrected by the addition of compounds during oenological practice (such as alcohol content, pH, total sulfur dioxide and methanol). Factors were extracted according to a minimum eigenvalue criterion of 1.0 and a varimax rotation was used.

**Figure 3:**
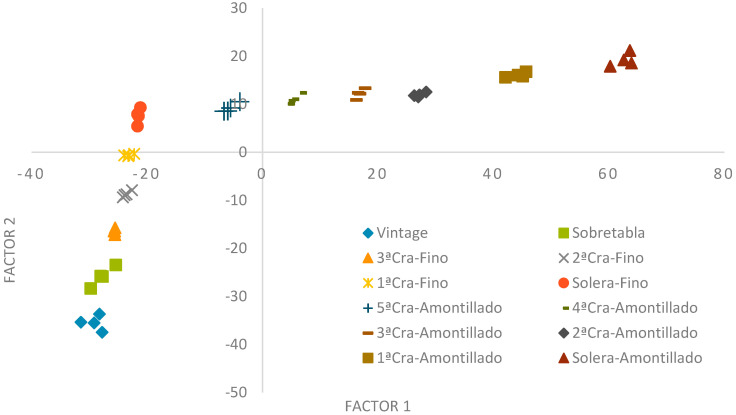
Projection of the wines on the plane of Factors 1 and 2 obtained after Principal Components analysis.

In the projection of the samples on the plane of Factors 1 and 2, two clear clusters can be observed (Figure 3). Firstly, there is a cluster of wines aged under biological aging (Sobretabla and Fino wines) and, secondly, there is a cluster of wines aged under oxidative aging (Amontillado wines). On the X-axis (Factor 1), the samples are ordered from left to right according to the age of the samples: vintage > Sobretabla > 3rd Criadera Fino > 2nd Criadera Fino > 1st Criadera Fino > Solera Fino > 5th Criadera Amontillado > 4th Criadera Amontillado > 3rd Criadera Amontillado > 2nd Criadera Amontillado > 1st Criadera Amontillado > Solera Amontillado). Factor 1 is, therefore, related to aging. The Y-axis (Factor 2) shows an ordering of the samples according to their biological aging (earlier or later stages). Factor 2 is, therefore, related to the biological component. These factor orderings and influences are supported by the literature, as they have been observed in previous work carried out in the research group [15].

The loadings of Factors 1 and 2 after varimax rotation are shown in Table 9 (only those that are more significant, whose r > 0.8, are shown). Factor 1, related to aging, shows high and positive correlations with many of the variables studied, including total acidity and volatile acidity, which are closely related to aging since, as time goes by, the aged wine is enriched with wood acids and favors the concentration of those it already had and those acquired due to the *merma* effect. Succinic and lactic acids also owe their correlation with aging to their concentration due to the *merma* effect. Total dry extract and total reducing atter are also high in older wines, as a consequence of the transfers by the wood and the *merma* concentrations of all the compounds that define both. Absorbance at 470 nm has turned out to be another important variable; as the wine ages, it acquires a tone ranging from yellow, gold, old gold, topaz, to intense amber in those of greater aging and, therefore, the value of this Absorbance increases. The fact that wood provides mostly phenolic compounds explains why many of the phenolic compounds determined have positive and high correlations with Factor 1 as well as FCI. Caffeic acid was the only variable that showed a high and negative correlation with Factor 1, as a consequence of the hydrolytic and oxidation reactions that take place and cause this compound to disappear over time. In short, the highest correlations with Factor 1 are given by those variables that are closely related to aging, either because they come from the wood (the longer the contact time with the wine, the greater the transfer) or because their concentration is affected by the *merma* (the older the wine, the greater the *merma)* (Table 3).

Factor 2, related to biological aging, shows high and positive correlations with acetaldehyde, its acetal and acetoin, which are three of the compounds that are closely related to yeast metabolism and which increase in concentration during biological aging as a consequence of being produced by yeast. The increase of gallic acid in the wines is due to transfers from the wood. In addition, high and negative correlations have been observed with citric, tartaric and malic acids and with glycerol. Glycerol is used by yeasts as a carbon source in their metabolism and the organic acids mentioned are also involved in the various metabolic pathways. Therefore, the variables that have the greatest weight in Factor 2 extracted from the model are those that are closely related to biological activity, confirming the conclusions drawn from Figure 3.

Factors 3, 4 and 5 are not as relevant since Factors 1 and 2 explain 92.3% of the total variability of the data explained by the model. The analysis of the factor loading matrix confirms the interpretation of the projection of the samples on the plane of Factors 1 and 2 (Figure 3), making it possible to establish a differentiating profile between the different wines, their age and type of aging. 

### 3.8. Multiple Linear Regression Analysis for Predicting the Age of Wines

The aim of the implemented chemometric study was to look for those variables studied that have a direct relationship with the age of the wine and that can serve as indicators of age, thus confirming the recorded age of the wines. The statistical technique used for this purpose was Multiple Linear Regression (MLR) and the strategy followed was to search for a model with as few independent variables as possible, limited to 5. 

For this purpose, a first study was carried out grouping the variables by families of compounds: organic acids and glycerol, volatile compounds (alcohols and higher esters), phenolic compounds and other variables (sulfates, phosphates, potassium, calcium, total dry extract, total reducing matter and absorbance at 470 nm). The StatGraphics 19 procedure “Multiple Regression Models” was used to find the best model for each group of variables. This procedure combines all predictor variables from 0 to 5 and calculates the statistics for their evaluation, among which are the mean square error (MSE), the R^2^ values adjusted for degrees of freedom (DF), Mallows’ Cp, *p*-values of the predictor variables and of the model at 95% significance and the Schwarz-Bayesian information criterion (SBIC). To select the best model, in all cases, it was required that the *p*-values of the predictor variables and the model were less than 0.05, the R^2^ as close to 100 as possible, and the Mallows and SBIC Cp criteria better (smaller values). The models constructed with four independent variables were the ones that yielded the best results in the cases studied.

Once the best model had been obtained for each group of variables, the possibility of combining the different groups of variables were tested and the same strategy was followed but taking as predictor variables to be explored only those variables that had been previously selected in the previous models. On the other hand, and given that the samples correspond to two different *Criaderas y Solera* systems (one of Fino wine, which is subjected only to Biological Aging, and another of Amontillado wine), where biological and oxidative aging converge, it was decided to study the two systems separately. In the Fino and Amontillado wines, five cases were extracted from each of the corresponding data matrices and used to validate the model once it was built.

#### 3.8.1. Determination of the Average Age of Fino Wines during the Aging Process

Table 10 shows the best models found for the Fino wine samples. As can be seen, the F5 model combining all the variables, previously selected, is a model with a significance level above 95% and with an R^2^ (adjusted for DF) that explains 99.9111% of the variance. The four variables that are part of the equation of the F5 model are closely linked to the microbiological and physicochemical behavior of biological aging. Glycerol is a carbon source used by the yeasts of the veil of flor yeasts for their development and metabolism, as well as an indicator of the better or worse biological aging that the wine has undergone during its aging; so much so, that values of less than 1 g/L in the Solera are evidence of the constant presence of a veil of flor yeasts on the surface of the Fino wine. Acetaldehyde comes mainly from the metabolic action of the yeasts of the veil of flor, which produce it and is responsible for the characteristic aromas of green apple and almonds that stand out organoleptically in this wine (Section 3.9). In the defined equation, the variable glycerol has a negative sign, while acetaldehyde has a positive sign, in the same way, that the value of glycerol decreases with biological aging and, on the contrary, Acetaldehyde increases with aging. Potassium is strongly influenced by the precipitation of potassium bitartrate in the early stages of aging, mainly due to the oversaturation of this salt in the youngest Sobretabla and Criaderas, its relatively low solubility in this hydroalcoholic medium and the lower temperatures in winter in the *Criaderas y Solera* systems. Syringic acid, derived from wood lignin, increases with aging, indicating its relationship with the length of time the wine remains in the oak casks. In the defined equation, these variables add negative or positive signs depending on whether they decrease or increase with age, as discussed in the previous sections. 

To validate this model, the cases that had previously been extracted from the sample set were submitted, the result is shown in Table 11. The accuracy of the model is high; obviously, there is a higher error in the younger samples, but from three years of age onwards, accuracy increases considerably.

#### 3.8.2. Determination of the Average Age of Amontillado Wines during the Aging Process

In the case of the Amontillado wines, we proceeded in the same way as in the Fino wines. Table 12 shows the best models obtained for each of the families of compounds. As in the previous case, the models have a high level of significance and accuracy. The four variables present in the equation defined by the global model are related to the physicochemical of the oxidative aging of Amontillado. 

Vanillic acid, extracted directly from the lignin of oak casks or through the oxidation of its aldehyde (vanillin), increases in concentration with age. Diethyl succinate, resulting from the esterification of succinic acid with ethanol, increases its value significantly during aging. This is due to the higher concentration of succinic acid with aging due to the effect of the *merma*, being detected as the main organic acid in the oldest Amontillado, as well as the significant increase in alcoholic strength that the wine acquires due to the same effect of the *merma*. Potassium increases in concentration with aging due to the effect of *merma*, despite the precipitation of potassium bitartrate that continues to occur due to the increase of alcoholic strength in the wine and is present in a supersaturated form in the wine. Sulfates also increase their concentration with *merma*, but to a lesser extent than expected, and it can be considered that this anion is partly insolubilized as calcium sulfate due to its low solubility as the alcohol content of the Amontillado wine increases. In the equation, the first three compounds give a positive sign in the formula, while that of sulfate is negative as it has lower values than expected.

In the same way, it was validated using a set of samples drawn from the initial set Table 13. As can be seen, the accuracy of the model is high, although there is obviously a greater error at the ends, but in no case does it reach errors of more than one year.

### 3.9. Tasting Sessions of Fino and Amontillado Sherry Wines

Table 14 shows the mean scores given by the panel to the four samples evaluated, as well as the standard deviations, which are less than 1 in all cases, supporting the homogeneity of the panel.

The *p*-values of the analysis of variance applied to the data are shown in Table 14. The significant increase in the intensities of the olfactory and olfactory-gustatory descriptors from the young fortified wine sample to any of the aged wines is confirmed. Yet, other significant differences occur as well.

The aromatic intensity and the olfactory notes of nuts and oak were perceived significantly better in the older Amontillado wine than in the younger Amontillado and the Fino wine, and that wine also presented the highest scores in balance and persistence. Of these descriptors, the olfactory intensity and the olfactory-gustatory ones were perceived as similar in Fino and 12-year-old Amontillado. However, the smell of nuts was somewhat greater in the Fino wine than in the younger Amontillado, and the notes of both wines differed in their quality: Fino was associated with almonds and Amontillado, with hazelnuts. 

**Figure 4:**
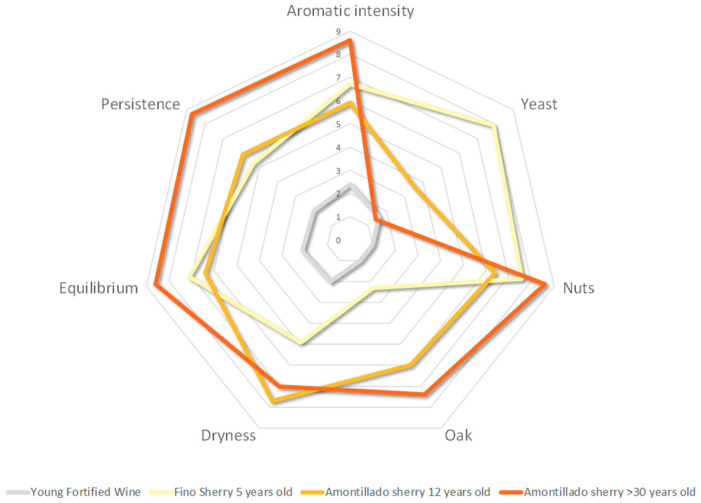
Spider graph of the average scores of the tasting panel for the wines evaluated.

On the other hand, the Fino wine was distinguished by an intense yeast smell, a note that still appears, although attenuated, in the younger Amontillado, as a “memory” of that first stage of biological aging. Finally, the oak and dryness in the mouth are perceived more intensely in the Amontillado wines, although only the oak scores higher with age. The spider graph (Figure 4) shows these differences.

**Figure 5:**
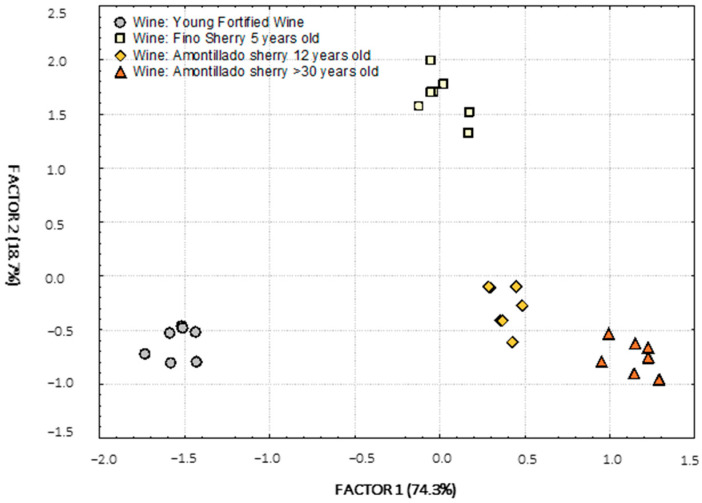
Projection of the wines on the plane of Factors 1 and 2 obtained after factor analysis.

When the analysis is approached from a multivariate perspective, the results are similar. In the application of the factor analysis with the extraction of factors by means of principal components, two factors were obtained that explain 93% of the total variability of the data. After a varimax rotation, the loadings (Table 14) allow an interpretation of the factors according to which Factor 1 is related to the barrel aging process (positive and high correlations, r > 0.88, with aromatic intensity, dried fruits, oak, dryness, balance and persistence) while Factor 2 shows a strong correlation (r > 0.98) with yeast odor, which relates it to biological aging under a veil of flor yeasts. Factors 1 and 2 of the Factor Analysis carried out in Section 3.7 also correlate with old age and biological aging, respectively. The projection of the samples on the plane of Factors 1 and 2 (Figure 5), in addition to the verification of the convergence of the tasting panel’s scores for each wine, i.e., their homogeneity, makes it possible to confirm the interpretation of the factors and establish a differentiating profile between the different wines. Regarding the horizontal axis Factor 1, the wines located from left to right (young fortified, Fino, Amontillado 12 years old, Amontillado >30 years old) are ordered according to their average aging time, the time they have been in contact with the casks, which generally gives them, except for slight inversions, higher intensities in 6 of the 7 descriptors analyzed, i.e., aromatic intensity, dried fruit and oak, dryness, balance and persistence. Regarding Factor 2, related to biological aging, the sample that shows the highest values and is completely different is the Fino wine, followed at a distance by Amontillado 12 years old, young wine and Amontillado >30 years old. This is not surprising, considering that Amontillado wines began with a biological aging stage, the aromatic character of which is attenuated by the oxidative aging to which they are subsequently subjected. Meanwhile, it is normal for a young, recently fermented wine to have a certain yeasty odor.

## 4. Conclusions

Very marked differences between Fino and Amontillado wines as a consequence of biological and oxidative aging have been shown. These differences are mainly due to the metabolism of the flor yeasts and their biological activity during the aging of the Fino wine. This biological activity, which after 5 years of aging begins to decrease naturally, is completely interrupted with the fortification of the Solera of Fino, giving way to the *soleraje* stage of Amontillado and marking a change in the behavior of the parameters analyzed in these Sherry wines. Under the oxidative environment, the contributions of the wood and the concentrations of the compounds due to the *merma*, as well as oxidation reactions, start to gain relevance. All this has a great impact on the organoleptic characteristics of the Sherry wines, as could be seen in the tasting sessions carried out, in which the particularities of each one of them stand out.

These trends are reflected in the chemometric study carried out with Cluster Analysis and Factor Analysis, in which clear clusters of wines aged under biological aging and in an oxidative environment are observed, in addition to the age of the wines. The Multiple Linear Regression study carried out shows a strong correlation between the age of Sherry wines and the parameters determined, arriving at two different models, one for Fino and the other for Amontillado wines, in which, with only four variables in each of them, the average age of these wines can be estimated with more than 99% confidence. The variables that were finally selected have shown a clear relationship with the type of aging that the wine has undergone (biological or oxidative), as well as with the contributions of the wood, since the longer the contact time, the greater the transfer of compounds from the cask. This type of model can be very useful in the industry, because with the determination of only four parameters, the ages of these wines can be estimated, either with the purpose of knowing such data or as a methodology tool for their quality control.

## Figures and Tables

**Table 1:** Nose and mouth descriptors and patterns used during the work with the tasters.

Descriptor	Definition	Pattern
*Odour*		
Aromatic intensity	Intensity of all the positive aromatic notes of the wine.	Amontillado wine with 50 years old of average aging.
Yeast	Aromas of fresh bread dough, characteristic of organic aging.	Fino wine with 5 years old of medium aging, extracted from casks with an intense white flor yeast development and clear hints of pungency (acetaldehyde and almond).
Nuts	Nut aromas, mainly almond in Fino wines and hazelnut and other nuts in Amontillado wines.	The aromatic intensity pattern for Amontillado and the yeast pattern for Fino wines.
Oak	Characteristic aromas of oak, with hints of dried fruits and vanilla.	Amontillado wine with 50 years old of average aging.
*Flavor*		
Dryness	Dry sensation of the wine, without astringency on the palate.	Amontillado wine with 20 years old of average aging.
Equilibrium	Overall positive evaluation of the sensations in the mouth, with a good integration of the acidity, without astringency or harshness, but with the aromatic reminder of the oak, as appropriate for an oak-aged wine.	The aromatic intensity pattern for Amontillados and the yeast pattern for Fino wines.
Persistence	Time evaluation of the positive olfactory-gustatory notes remaining after the final sip.	The aromatic intensity pattern for Amontillado and the yeast pattern for Fino wines.

**Table 2:** Oenological control parameters: alcoholic strength (%vol.), density (g/L), pH, total acidity (g TH_2_/L), volatile acidity (g AcH/L), glycerol (mg/L), total sulfur dioxide (mg/L), sulfates (g K_2_SO_4_/L), phosphates (mg PO_4_/L), potassium (mg/L), calcium (mg/L), total dry extract (g/L), reducing substances (g/L) y sugar-free extract (g/L) in the studied Sobretabla, Fino and Amontillado wines.

	Vintage	Sobretabla	3rd Cra F	2nd Cra F	1st Cra F	Solera F	5th Cra A	4th Cra A	3rd Cra A	2nd Cra A	1st Cra A	Solera A
Alcoholic strength	15.63 ± 0.15 ^a^	15.10 ± 0.26 ^b,c^	15.17 ± 0.15 ^a^	15.27 ± 0.15 ^a^	15.10 ± 0.20 ^a^	14.97 ± 0.15 ^a^	17.10 ± 0.20 ^d^	17.63 ± 0.21 ^e^	18.43 ± 0.06 ^a^	19.17 ± 0.06 ^g^	19.87 ± 0.06 ^a^	20.63 ± 0.12 ^i^
Density	986.1 ± 0.3 ^a^	986.3 ± 0.5 ^a^	985.6 ± 0.3 ^b^	985.1 ± 0.2 ^c^	984.5 ± 0.2 ^d^	984.1 ± 0.2 ^e^	983.3 ± 0.2 ^f^	984.1 ± 0.1 ^d,e^	984.3 ± 0.1 ^d,e^	984.5 ± 0.1 ^d^	985.3 ± 0.1 ^b,c^	985.8 ± 0.1 ^a,b^
pH	3.20 ± 0.05 ^a,c^	3.15 ± 0.07 ^a,b,c^	3.14 ± 0.03 ^b,c^	3.12 ± 0.02 ^b,d^	3.09 ± 0.01 ^d^	3.09 ± 0.02 ^d^	3.19 ± 0.01 ^c,e^	3.22 ± 0.02 ^a^	3.24 ± 0.01 ^a,f^	3.27 ± 0.01 ^f^	3.29 ± 0.02 ^f^	3.29 ± 0.02 ^f^
Total acidity	5.99 ± 0.23 ^a^	5.30 ± 0.18 ^b^	4.91 ± 0.12 ^c^	4.78 ± 0.10 ^c^	4.41 ± 0.04 ^d^	4.12 ± 0.03 ^e^	4.93 ± 0.08 ^c^	5.59 ± 0.13 ^f^	6.40 ± 0.03 ^g^	6.93 ± 0.10 ^h^	7.30 ± 0.06 ^i^	7.71 ± 0.17 ^j^
Volatile acidity	0.25 ± 0.08 ^a^	0.29 ± 0.08 ^a,c^	0.30 ± 0.04 ^a,c^	0.26 ± 0.02 ^a^	0.23 ± 0.06 ^a^	0.15 ± 0.02 ^b^	0.34 ± 0.04 ^c^	0.48 ± 0.02 ^d^	0.58 ± 0.02 ^e^	0.66 ± 0.02 ^f^	0.75 ± 0.02 ^g^	0.88 ± 0.02 ^h^
Total sulfur dioxide	67 ± 11 ^a^	51 ± 10 ^b^	44 ± 7 ^b,c^	39 ± 3 ^c,d^	31 ± 3 ^d,e^	23 ± 2 ^e^	14 ± 2 ^f^	9 ± 1 ^f,e^	5 ± 1 ^e^	4 ± 1 ^e^	1 ± 1 ^e^	1 ± 1 ^e^
Sulfates	1.27 ± 0.23 ^a^	1.19 ± 0.06 ^a,b^	1.12 ± 0.03 ^b,c^	1.05 ± 0.02 ^b,c,d^	0.98 ± 0.03 ^c,d^	0.96 ± 0.02 ^d^	1.17 ± 0.03 ^a,c,d^	1.27 ± 0.05 ^a^	1.49 ± 0.04 ^e^	1.76 ± 0.04 ^f^	1.95 ± 0.10 ^g^	2.23 ± 0.04 ^h^
Phosphates	85.33 ± 16.26 ^a^	76.00 ± 6.56 ^b^	70.00 ± 2.65 ^b,c^	66.67 ± 2.52 ^c^	61.33 ± 1.53 ^c,d^	57.67 ± 1.53 ^d^	60.67 ± 2.08 ^d^	66.67 ± 2.52 ^c,d^	77.67 ± 1.53 ^a,b^	86.33 ± 2.08 ^a^	98.33 ± 2.52 ^e^	116.67 ± 2.52 ^e^
Potassium	1136 ± 43 ^a^	881 ± 33 ^b^	792 ± 9 ^c^	772 ± 6 ^c^	784 ± 14 ^c^	802 ± 6 ^c^	1091 ± 13 ^e^	1201 ± 9 ^f^	1379 ± 10 ^g^	1658 ± 12 ^h^	2142 ± 9 ^i^	2575 ± 60 ^j^
Calcium	110 ± 21 ^a,c^	106 ± 16 ^a,b,c^	99 ± 3 ^a,b^	99 ± 3 ^a^	97 ± 2 ^a^	95 ± 2 ^b^	97 ± 3 ^a,b^	101 ± 3 ^a,b^	104 ± 3 ^a,b,c^	108 ± 2 ^a,b,c^	115 ± 4 ^c,d^	125 ± 3 ^d^
Glycerol	7151 ± 711 ^a^	5136 ± 548 ^b^	3641 ± 131 ^c^	2171 ± 68 ^d^	1176 ± 81 ^e,g^	611 ± 59 ^f^	1097 ± 90 ^e^	1290 ± 30 ^e,g^	1595 ± 78 ^g^	2075 ± 48 ^d^	2650 ± 60 ^h^	3741 ± 183 ^c^
Total dry extract	20.17 ± 0.40 ^a^	19.13 ± 0.42 ^b^	17.63 ± 0.40 ^c^	16.63 ± 0.40 ^d^	14.50 ± 0.79 ^e^	13.10 ± 0.17 ^f^	17.07 ± 0.12 ^c,d^	20.60 ± 0.70 ^a^	23.40 ± 0.17 ^g^	26.10 ± 0.00 ^h^	30.23 ± 0.25 ^i^	33.50 ± 0.35 ^j^
Reducing substances	2.63 ± 0.49 ^a^	1.10 ± 0.20 ^b^	0.80 ± 0.10 ^b,c^	0.73 ± 0.06 ^c^	0.70 ± 0.10 ^c^	0.60 ± 0.10 ^c^	1.63 ± 0.12 ^d^	2.27 ± 0.06 ^e^	2.83 ± 0.15 ^a,f^	3.03 ± 0.21 ^f^	3.53 ± 0.23 ^g^	4.90 ± 0.26 ^h^
Sugar-free extract	17.53 ± 0.23 ^a^	18.03 ± 0.61 ^a,b^	16.83 ± 0.38 ^a^	15.90 ± 0.46 ^c^	13.80 ± 0.78 ^d^	12.50 ± 0.10 ^e^	15.43 ± 0.12 ^c^	18.33 ± 0.68 ^b^	20.57 ± 0.21 ^f^	23.07 ± 0.21 ^g^	26.70 ± 0.46 ^h^	28.60 ± 0.56 ^i^

Mean values ± standard deviation (n = 4) are shown; ANOVA: different letters (a, b, c, d, e, f, g, h, i, j) indicate significant differences (*p* < 0.05). Vintage: Young fortified wine; Cra: Criadera; F: Fino; A: Amontillado.

**Table 3:** Effect of a *merma* of 3% in volume with aging for the different average aging times studied. Data extracted from the reference [33].

Average Age (Years Old)	Volume of Wine in the Casks (%)	Concentration Increase of Non-Volatile Compounds (%)
1	97.00	1.03
2	94.09	1.06
3	91.27	1.10
4	88.53	1.13
5	85.87	1.16
8	78.37	1.28
12	69.38	1.44
16	61.43	1.63
20	54.38	1.84
30	40.10	2.49
40	29.57	3.38

**Table 4:** Organic acids (mg/L) in the studied Sobretabla, Fino and Amontillado wines.

	Vintage	Sobretabla	3rd Cra F	2nd Cra F	1st Cra F	Solera F	5th Cra A	4th Cra A	3rd Cra A	2nd Cra A	1st Cra A	Solera A
Citric acid	244 ± 67 ^a^	177 ± 41 ^b^	146 ± 0 ^b,c,d^	128 ± 7 ^c,e^	101 ± 4 ^e,f^	75 ± 8 ^f^	88 ± 9 ^f^	94 ± 4 ^e,f^	100 ± 2 ^e,f^	106 ± 4 ^e,f^	108 ± 5 ^d,e,f^	113 ± 5 ^d,e,f^
Tartaric acid	5121 ± 439 ^a^	3809 ± 161 ^b^	3024 ± 63 ^c^	2782 ± 53 ^d^	2490 ± 26 ^e^	2139 ± 47 ^f^	1599 ± 71 ^g^	1430 ± 36 ^g,h^	1193 ± 31 ^h,i^	1235 ± 36 ^h,i^	1046 ± 21 ^i,j^	951 ± 36 ^j^
Malic acid	491 ± 116 ^a^	385 ± 54 ^b^	105 ± 7 ^c^	32 ± 9 ^d^	12 ± 5 ^d^	4 ± 3 ^d^	15 ± 2 ^d^	20 ± 3 ^d^	22 ± 3 ^d^	26 ± 3 ^d^	47 ± 4 ^c,d^	65 ± 5 ^d^
Succinic acid	641 ± 82 ^a^	573 ± 86 ^b^	507 ± 22 ^c^	428 ± 9 ^d^	397 ± 4 ^d^	381 ± 6 ^d^	502 ± 18 ^c^	603 ± 18 ^a,b^	728 ± 8 ^e^	847 ± 20 ^f^	1028 ± 18 ^g^	1246 ± 15 ^h^
Lactic acid	267 ± 71 ^a^	301 ± 27 ^a,b^	479 ± 10 ^c^	413 ± 9 ^d^	213 ± 11 ^e^	116 ± 7 ^f^	340 ± 14 ^b^	462 ± 14 ^c^	582 ± 13 ^g^	703 ± 20 ^h^	855 ± 12 ^i^	1016 ± 40 ^j^

Mean values ± standard deviation (n = 4) are shown; ANOVA: different letters (a, b, c, d, e, f, g, h, i, j) indicate significant differences (*p* < 0.05). Vintage: Young fortified wine; Cra: Criadera; F: Fino; A: Amontillado.

**Table 5:** Acetaldehyde, acetaldehyde-diethylacetal, acetoin, ethyl acetate, methanol, higher alcohols, ethyl esters of organic acids and ethyl esters of fatty acids in mg/L in the studied Sobretabla, Fino and Amontillado wines.

	Vintage	Sobretabla	3rd Cra F	2nd Cra F	1st Cra F	Solera F	5th Cra A	4th Cra A	3rd Cra A	2nd Cra A	1st Cra A	Solera A
Acetaldehyde	72.3 ± 19.0 ^a^	113.3 ± 21.8 ^b^	178.0 ± 11.4 ^c^	237.3 ± 9.6 ^d^	369.3 ± 14.2 ^e^	487.0 ± 14.5 ^f^	256.3 ± 12.2 ^d^	220.7 ± 3.5 ^d,g^	197.7 ± 4.2 ^c,h^	198.0 ± 3.6 ^c,h^	203.3 ± 5.7 ^g,h^	192.7 ± 9.1 ^c,h^
Acetaldehyde-dietilacetal	7.0 ± 2.6 ^a^	11.0 ± 2.0 ^a^	18.0 ± 2.0 ^b^	25.0 ± 1.0 ^c^	38.0 ± 1.7 ^d^	50.3 ± 2.1 ^e^	35.3 ± 5.9 ^d,f^	31.3 ± 2.1 ^f,g^	28.7 ± 2.1 ^c,g^	28.7 ± 1.5 ^c,g^	32.7 ± 4.2 ^f,g^	29.7 ± 3.1 ^c,g^
Acetoin	4.0 ± 1.0 ^a^	11.0 ± 3.0 ^b^	25.3 ± 2.1 ^c^	37.3 ± 1.5 ^d^	54.0 ± 3.6 ^e^	78.3 ± 2.5 ^f^	69.7 ± 4.0 ^g,i^	63.7 ± 2.1 ^h,i^	60.7 ± 1.2 ^h^	60.0 ± 2.6 ^h^	66.7 ± 2.5 ^g,i^	65.7 ± 1.5 ^g,i^
Ethyl acetate	49.0 ± 6.1 ^a^	61.3 ± 10.5 ^b^	56.3 ± 4.2 ^a,b^	44.7 ± 3.1 ^a,d^	37.3 ± 1.5 ^d^	36.7 ± 4.0 ^d^	107.0 ± 7.0 ^e^	150.3 ± 4.7 ^f^	173.3 ± 4.5 ^g^	203.0 ± 4.6 ^h^	241.7 ± 4.0 ^i^	282.0 ± 5.6 ^j^
Methanol	65.7 ± 7.4 ^a,b^	62.7 ± 9.0 ^a^	61.7 ± 3.1 ^a^	66.7 ± 1.5 ^a,b^	67.0 ± 3.0 ^a,b^	71.0 ± 1.0 ^b,c^	78.0 ± 3.0 ^c^	88.0 ± 3.6 ^d^	97.3 ± 3.5 ^e^	101.0 ± 3.0 ^e,f^	104.7 ± 2.1 ^f^	117.3 ± 4.5 ^g^
Higher alcohols												
N-Propanol	30.0 ± 2.6 ^a^	35.0 ± 5.3 ^b^	37.3 ± 2.5 ^c^	41.3 ± 1.5 ^c^	45.7 ± 3.1 ^d^	46.3 ± 1.5 ^d^	50.7 ± 1.5 ^e^	54.7 ± 2.5 ^e,f^	57.3 ± 2.1 ^f^	61.7 ± 1.2 ^g^	67.7 ± 1.5 ^h^	72.7 ± 1.5 ^i^
Isobutanol	37.7 ± 4.5 ^a^	44.7 ± 1.2 ^b^	47.7 ± 1.2 ^b,c^	51.3 ± 0.6 ^c,d^	53.3 ± 1.2 ^d^	55.0 ± 1.0 ^d^	67.0 ± 1.7 ^e^	69.7 ± 1.5 ^e,f^	72.7 ± 1.5 ^f^	77.0 ± 3.0 ^g^	80.0 ± 3.6 ^g^	86.3 ± 1.5 ^h^
2-Methyl-1-butanol	40.0 ± 7.2 ^a^	41.3 ± 4.0 ^a,b^	44.3 ± 2.5 ^a,b,c^	46.3 ± 2.1 ^b,c^	48.7 ± 2.1 ^c,d^	54.0 ± 2.6 ^d,e^	55.7 ± 2.5 ^e,f^	57.0 ± 1.7 ^f,g^	60.0 ± 2.0 ^f,g,h^	61.3 ± 1.2 ^g,h^	61.7 ± 2.1 ^g,h^	64.0 ± 4.0 ^h^
3-Methyl-1-butanol	154.3 ± 26.1 ^a^	167.3 ± 19.6 ^a,b^	172.0 ± 3.6 ^b^	182.3 ± 5.0 ^b,c^	191.0 ± 2.6 ^c,d^	205.0 ± 8.9 ^d,e^	219.3 ± 4.7 ^e,f^	227.7 ± 5.1 ^f,g^	233.0 ± 4.6 ^f,g^	235.7 ± 4.7 ^f,g^	237.0 ± 3.6 ^g^	242.3 ± 4.0 ^g^
Hexanol	1.17 ± 0.57 ^a^	0.83 ± 0.25 ^b^	0.73 ± 0.06 ^b^	0.70 ± 0.10 ^b^	0.77 ± 0.12 ^b^	0.70 ± 0.10 ^b^	0.80 ± 0.10 ^b^	0.77 ± 0.06 ^b^	0.77 ± 0.06 ^b^	0.73 ± 0.06 ^b^	0.80 ± 0.10 ^b^	0.87 ± 0.06 ^a,b^
2-Phenyl ethanol	16.87 ± 12.19 ^a^	18.20 ± 4.83 ^a,b^	19.90 ± 0.62 ^a,b^	20.60 ± 1.21 ^a,b,c^	20.93 ± 0.55 ^a,b,c^	21.37 ± 0.50 ^a,b,c,d^	23.90 ± 0.44 ^b,c,d,e^	26.57 ± 0.93 ^c,d,e,f^	27.57 ± 1.15 ^d,e,f^	29.23 ± 0.29 ^e,f,g^	31.13 ± 0.40 ^f,g^	34.40 ± 0.30 ^g^
Ethyl esters of organic acids												
Ethyl lactate	20.20 ± 3.97 ^a^	23.73 ± 0.90 ^b^	27.07 ± 0.35 ^b^	31.13 ± 0.31 ^c^	31.63 ± 0.70 ^c^	33.20 ± 0.95 ^c^	80.67 ± 1.27 ^d^	103.27 ± 2.18 ^e^	126.17 ± 4.77 ^f^	146.70 ± 0.72 ^g^	173.80 ± 0.30 ^h^	199.30 ± 2.05 ^i^
Diethyl succinate	1.10 ± 0.35 ^a^	4.77 ± 0.70 ^b^	12.07 ± 0.72 ^c^	14.80 ± 0.46 ^d^	16.27 ± 0.35 ^e^	18.50 ± 1.11 ^f^	52.77 ± 0.91 ^g^	59.17 ± 0.64 ^h^	65.20 ± 0.89 ^i^	70.33 ± 0.45 ^j^	81.73 ± 0.85 ^k^	93.93 ± 1.37 ^l^
Ethyl esters of fatty acids												
Ethyl caproate	0.23 ± 0.15 ^a,c^	0.27 ± 0.06 ^a,b,c^	0.20 ± 0.10 ^a^	0.30 ± 0.10 ^a,b,c^	0.33 ± 0.06 ^c,d^	0.37 ± 0.06 ^c,d^	0.33 ± 0.06 ^c,d^	0.37 ± 0.06 ^b,d^	0.33 ± 0.06 ^c,d^	0.33 ± 0.06 ^c,d^	0.43 ± 0.06 ^d^	0.47 ± 0.06 ^e^
Ethyl caprylate	0.57 ± 0.25 ^a^	0.53 ± 0.06 ^a^	0.67 ± 0.06 ^a,b^	0.70 ± 0.10 ^a,b^	0.87 ± 0.06 ^b,c^	1.07 ± 0.12 ^c^	4.03 ± 0.15 ^d^	4.40 ± 0.10 ^e^	4.67 ± 0.15 ^f^	4.90 ± 0.10 ^g^	5.23 ± 0.06 ^a^	5.50 ± 0.10 ^i^
Ethyl caprate	0.17 ± 0.12 ^a^	0.17 ± 0.06 ^a^	0.17 ± 0.06 ^a^	0.20 ± 0.10 ^a^	0.27 ± 0.06 ^a,b^	0.33 ± 0.06 ^b,c^	0.37 ± 0.06 ^b,c^	0.37 ± 0.06 ^b,c^	0.43 ± 0.06 ^c^	0.33 ± 0.06 ^b,c^	0.33 ± 0.06 ^b,c^	0.33 ± 0.06 ^b,c^
Ethyl laureate	0.13 ± 0.06	0.13 ± 0.06	0.17 ± 0.06	0.13 ± 0.06	0.17 ± 0.06	0.23 ± 0.06	0.23 ± 0.06	0.20 ± 0.10	0.23 ± 0.12	0.20 ± 0.10	0.20 ± 0.10	0.20 ± 0.10
Ethyl myristate	0.07 ± 0.06	0.03 ± 0.06	0.07 ± 0.06	0.07 ± 0.06	0.07 ± 0.06	0.10 ± 0.00	0.13 ± 0.06	0.10 ± 0.10	0.07 ± 0.06	0.10 ± 0.10	0.13 ± 0.06	0.13 ± 0.06
Ethyl palmitate	0.13 ± 0.06 ^a,b,c^	0.13 ± 0.06 ^a,b,c^	0.07 ± 0.06 ^a^	0.10 ± 0.10 ^a,c^	0.13 ± 0.06 ^a,b,c,d^	0.17 ± 0.06 ^a,b,c,d^	0.17 ± 0.06 ^a,b,c,d^	0.23 ± 0.06 ^b,c,d^	0.23 ± 0.06 ^b,c,d^	0.20 ± 0.10 ^c,d^	0.23 ± 0.06 ^d^	0.23 ± 0.06 ^d^

Mean values ± standard deviation (n = 4) are shown; ANOVA: different letters (a, b, c, d, e, f, g, h, i, j, k, l) indicate significant differences (*p* < 0.05). Vintage: Young fortified wine; Cra: Criadera; F: Fino; A: Amontillado.

**Table 6:** Percentage composition (%) of flor yeasts in veil present in the biological aging of the Fino and Sobretabla wine studied [48].

Wine Type	*Sacch. Cerevisiae* *(Race: Beticus)*	*Sacch. Cerevisiae* *(Race: Montuliensis)*	*Sacch. Cerevisiae* *(Race: Cheresiensis)*	*Sacch. Cerevisiae* *(Race: Rouxii)*
Sobretabla	100	0	0	0
3rd Criadera. Fino	91 ± 9	5 ± 2	4 ± 2	0
2nd Criadera. Fino	81 ± 11	11 ± 4	8 ± 4	0
1st Criadera. Fino	72 ± 8	19 ± 5	8 ± 2	1 ± 0
Solera Fino	58 ± 8	38 ± 9	3 ± 1	1 ± 0

Mean values ± standard deviation (n = 200) are shown. Samples were taken from 10 casks per year (4 years) and per wine type (5).

**Table 7:** Folin-Ciocalteau Index FCI (mg gallic acid/L) and concentration of phenolic compounds (mg/L) in the Sobretabla, Fino and Amontillado wines studied.

	Vintage	Sobretabla	3rd Cra F	2nd Cra F	1st Cra F	Solera F	5th Cra A	4th Cra A	3rd Cra A	2nd Cra A	1st Cra A	Solera A
FCI	246 ± 13 ^a^	265 ± 10 ^b^	282 ± 4 ^c^	293 ± 6 ^c,d^	300 ± 3 ^d^	306 ± 7 ^d^	377 ± 8 ^e^	414 ± 8 ^f^	436 ± 9 ^g^	455 ± 7 ^h^	469 ± 7 ^i^	534 ± 10 ^j^
Gallic acid	3.83 ± 0.31 ^a^	4.93 ± 0.54 ^b^	6.78 ± 0.68 ^c^	8.21 ± 0.2 ^d^	9.62 ± 0.21 ^e^	11.06 ± 0.31 ^f^	11.27 ± 0.23 ^f^	12.26 ± 0.26 ^g^	11.72 ± 0.27 ^g,f^	9.73 ± 0.55 ^e^	9.69 ± 0.73 ^e^	10.14 ± 0.79 ^e^
p-Hydroxybenzoic acid	0.24 ± 0.10 ^a^	0.46 ± 0.15 ^a^	0.47 ± 0.03 ^c^	0.54 ± 0.04 ^c,d^	0.59 ± 0.04 ^c,d^	0.61 ± 0.03 ^d^	0.77 ± 0.04 ^e^	0.86 ± 0.07 ^e^	1.03 ± 0.07 ^f^	1.25 ± 0.07 ^g^	1.82 ± 0.13 ^h^	1.94 ± 0.07 ^h^
Vanillic acid	0.09 ± 0.02 ^a^	0.32 ± 0.12 ^b^	0.50 ± 0.04 ^c^	0.49 ± 0.04 ^c^	0.62 ± 0.05 ^d^	0.63 ± 0.04 ^d^	1.01 ± 0.04 ^e^	1.42 ± 0.03 ^f^	1.86 ± 0.06 ^a^	2.31 ± 0.08 ^h^	3.29 ± 0.11 ^i^	4.76 ± 0.09 ^j^
Syringic acid	0.39 ± 0.14 ^a^	0.40 ± 0.08 ^a^	0.53 ± 0.04 ^a,b^	0.70 ± 0.03 ^b,c^	0.82 ± 0.04 ^c^	1.02 ± 0.04 ^d^	1.10 ± 0.06 ^d^	1.53 ± 0.03 ^e^	2.52 ± 0.05 ^f^	3.49 ± 0.07 ^g^	4.86 ± 0.21 ^h^	6.31 ± 0.25 ^i^
Protocathetic acid	0.31 ± 0.12 ^a^	0.53 ± 0.11 ^a^	2.04 ± 0.13 ^b^	3.24 ± 0.07 ^c^	3.71 ± 0.12 ^d^	3.53 ± 0.21 ^d^	3.63 ± 0.18 ^d^	3.81 ± 0.22 ^d,e^	4.04 ± 0.10 ^e,f^	4.32 ± 0.21 ^f^	5.03 ± 0.29 ^g^	6.08 ± 0.18 ^h^
Caffeic acid	4.97 ± 0.71 ^a^	4.43 ± 0.18 ^b^	3.92 ± 0.10 ^c^	3.74 ± 0.16 ^c^	3.60 ± 0.11 ^c^	3.00 ± 0.12 ^d^	2.90 ± 0.13 ^d^	2.00 ± 0.07 ^e^	0.84 ± 0.05 ^f^	0.82 ± 0.15 ^f^	0.58 ± 0.10 ^f,g^	0.33 ± 0.04 ^g^
trans-Caftaric acid	41.71 ± 13.83 ^a^	31.62 ± 2.50 ^b^	26.15 ± 0.76 ^b,c^	23.47 ± 0.65 ^c^	19.23 ± 1.24 ^c,d^	15.53 ± 1.15 ^d,e^	11.82 ± 0.94 ^e,f^	9.28 ± 0.53 ^e,f,g^	7.67 ± 0.30 ^f,g^	6.63 ± 0.43 ^f,g^	4.31 ± 0.21 ^g^	2.14 ± 0.34 ^g^
p-Coumaric acid	0.41 ± 0.10 ^a^	0.50 ± 0.08 ^b^	0.84 ± 0.07 ^c^	0.98 ± 0.04 ^c,d^	1.07 ± 0.05 ^d^	1.12 ± 0.03 ^d^	1.64 ± 0.04 ^e^	1.73 ± 0.02 ^e,f^	1.80 ± 0.08 ^f^	2.62 ± 0.12 ^g^	3.46 ± 0.19 ^h^	4.18 ± 0.07 ^i^
cis-Coutaric acid	5.65 ± 0.52 ^a^	5.31 ± 0.33 ^a,b^	5.03 ± 0.25 ^b^	3.94 ± 0.13 ^c^	3.24 ± 0.13 ^d^	2.90 ± 0.16 ^d^	1.65 ± 0.08 ^e^	1.42 ± 0.10 ^e,f^	1.10 ± 0.06 ^f,g^	0.81 ± 0.09 ^g^	1.02 ± 0.10 ^g^	1.10 ± 0.20 ^f,g^
trans-Coutaric acid	9.79 ± 2.13 ^a^	9.10 ± 0.57 ^a,b^	8.57 ± 0.17 ^b,c^	7.73 ± 0.27 ^c,d^	6.75 ± 0.15 ^d,e^	5.72 ± 0.26 ^e,f^	4.95 ± 0.10 ^f,g^	4.36 ± 0.13 ^g^	3.90 ± 0.13 ^g^	3.86 ± 0.11 ^g^	2.03 ± 0.10 ^g,h^	1.14 ± 0.10 ^h^
Ferulic acid	0.42 ± 0.13 ^a^	0.42 ± 0.03 ^a^	0.44 ± 0.04 ^a^	0.65 ± 0.04 ^b^	0.84 ± 0.05 ^c^	0.88 ± 0.07 ^c,d^	1.02 ± 0.03 ^e^	0.96 ± 0.02 ^d,e^	0.72 ± 0.01 ^b^	0.55 ± 0.04 ^f^	0.47 ± 0.04 ^a,f^	0.54 ± 0.08 ^f^
Fertaric acid	7.56 ± 1.19 ^a^	5.83 ± 0.45 ^b^	4.26 ± 0.10 ^c^	3.77 ± 0.09 ^c,d^	3.47 ± 0.06 ^d^	2.83 ± 0.09 ^e^	2.54 ± 0.09 ^e,f^	2.14 ± 0.06 ^f,e^	1.71 ± 0.04 ^e,f^	1.43 ± 0.06 ^a^	1.09 ± 0.04 ^f,g^	0.74 ± 0.08 ^g^
p-Hydroxybenzaldehyde	0.16 ± 0.08 ^a^	0.28 ± 0.09 ^a,b^	0.28 ± 0.05 ^a,b^	0.39 ± 0.02 ^b^	0.45 ± 0.05 ^b^	0.48 ± 0.04 ^b^	0.98 ± 0.05 ^c^	1.33 ± 0.07 ^d^	1.73 ± 0.07 ^e^	2.19 ± 0.16 ^f^	3.24 ± 0.26 ^g^	4.33 ± 0.27 ^h^
Vanillin	0.13 ± 0.04 ^a^	0.12 ± 0.04 ^a^	0.13 ± 0.02 ^a^	0.14 ± 0.02 ^a^	0.20 ± 0.02 ^a,b^	0.27 ± 0.04 ^b^	0.44 ± 0.03 ^c^	0.81 ± 0.03 ^d^	1.55 ± 0.02 ^e^	1.84 ± 0.12 ^f^	2.40 ± 0.10 ^g^	3.03 ± 0.15 ^h^
Syringaldehyde	0.39 ± 0.10 ^a^	0.40 ± 0.08 ^b^	0.68 ± 0.04 ^c^	0.79 ± 0.05 ^c,d^	0.82 ± 0.04 ^c,d^	0.88 ± 0.04 ^d^	1.55 ± 0.03 ^e^	2.42 ± 0.06 ^a^	3.33 ± 0.07 ^a^	4.73 ± 0.17 ^h^	6.36 ± 0.19 ^i^	8.04 ± 0.18 ^j^
5-Hydroxymethylfurfural	0.74 ± 0.09 ^a^	0.94 ± 0.08 ^a^	1.06 ± 0.07 ^a^	1.17 ± 0.08 ^a^	1.05 ± 0.16 ^a^	0.97 ± 0.11 ^a^	4.08 ± 0.30 ^b^	8.27 ± 0.37 ^c^	14.36 ± 0.48 ^d^	18.26 ± 1.31 ^e^	28.37 ± 2.00 ^f^	38.62 ± 1.47 ^g^
Furfural	0.09 ± 0.04 ^a^	0.09 ± 0.02 ^a^	0.11 ± 0.02 ^a^	0.12 ± 0.03 ^a^	0.13 ± 0.03 ^a^	0.16 ± 0.02 ^a^	2.54 ± 0.13 ^b^	4.07 ± 0.09 ^c^	6.59 ± 0.32 ^d^	10.03 ± 0.39 ^e^	14.56 ± 0.46 ^f^	18.96 ± 0.71 ^g^

Mean values ± standard deviation (n = 4) are shown; ANOVA: different letters (a, b, c, d, e, f, g, h, i, j) indicate significant differences (*p* < 0.05). Vintage: Young fortified wine; Cra: Criadera; F: Fino; A: Amontillado.

**Table 8:** Absorbance values at 470 nm (a.u.), related to the brownish-yellow/brownish tone, in the Sobretabla, Fino and Amontillado wines studied.

	Vintage	Sobretabla	3rd Cra F	2nd Cra F	1st Cra F	Solera F	5th Cra A	4th Cra A	3rd Cra A	2nd Cra A	1st Cra A	Solera A
A470	0.076 ± 0.003 ^a^	0.081 ± 0.003 ^a,b^	0.090 ± 0.003 ^b,c^	0.094 ± 0.003 ^c,d^	0.102 ± 0.003 ^d,e^	0.112 ± 0.004 ^e^	0.202 ± 0.010 ^f^	0.272 ± 0.006 ^g^	0.324 ± 0.008 ^h^	0.374 ± 0.009 ^i^	0.479 ± 0.006 ^j^	0.653 ± 0.013 ^k^

Mean values ± standard deviation (n = 4) are shown; ANOVA: different letters (a, b, c, d, e, f, g, h, i, j, k) indicate significant differences (*p* < 0.05). Vintage: Young fortified wine; Cra: Criadera; F: Fino; A: Amontillado.

**Table 9:** Coefficients of Factors 1 and 2 after varimax rotation of the variables that have shown the highest correlation (r > 0.8).

Variables	Factor 1	Factor 2
Total acidity (g tartaric acid/L)	0.949518	
Volatile acidity (g acetic acid/L)	0.958833	
Citric acid (mg/L)		−0.921827
Tartaric acid (mg/L)		−0.790403
Malic acid (mg/L)		−0.928412
Succinic acid (mg/L)	0.982632	
Lactic acid (mg/L)	0.961339	
Glycerol (mg/L)		−0.959727
Sulfate (g K_2_SO_4_/L)	0.984878	
Phosphate (mg PO_4_/L)	0.911762	
Potassium (mg/L)	0.975139	
Total dry extract(g/L)	0.985320	
Total reducing matter (g/L)	0.921695	
Absorbance 470 nm (a.u.)	0.946796	
Acetaldehyde (mg/L)		0.798644
Acetal (mg/L)		0.930717
Acetoin (mg/L)		0.922805
Ethyl acetate (mg/L)	0.943450	
n-Propanol (mg/L)	0.808014	
2-Phenyl ethanol (mg/L)	0.796238	
Ethyl lactate (mg/L)	0.916330	
Diethyl succinate (mg/L)	0.833209	
FCI (mg gallic acid/L)	0.852561	
Gallic acid (mg/L)		0.918513
p-Hydroxybenzoic ac. (mg/L)	0.922433	
Vanillic acid (mg/L)	0.949567	
Syringic acid (mg/L)	0.958999	
Caffeic acid (mg/L)	−0.796829	
p-Coumaric acid (mg/L)	0.917132	
Vanillin (mg/L)	0.962484	
Syringaldehyde (mg/L)	0.963981	
5-Hydroxymethylfurfural(mg/L)	0.974553	
Furfural (mg/L)	0.974096	

**Table 10:** Regression models (MLR) to estimate the aging of Fino wines. F1: organic acids and glycerol model; F2: polyphenols model; F3: volatile compounds model; F4: “other variables” model; F5: overall model.

Model	Regression	R^2^ (Adjusted for DF)	*p*-Value Model (95%)
F1	Average age (years old) = 5.7595 − 0.00398154 * Citric acid (mg/L) − 0.0023204 * Lactic acid (mg/L) − 0.00059102 * Glycerol (mg/L)	98.7826	0.0000
F2	Average age (years old) = −1.17355 + 0.283517 * Gallic acid (mg/L) + 1.43392 * p-Hydroxybenzoic acid (mg/L) + 2.61478 * Syringic acid (mg/L) − 0.0309252 * trans-Caftaric acid	99.77145	0.0000
F3	Average age (years old) = −4.13837 + 0.00564545 * Acetaldehyde (mg/L) + 0.0351109 * n-Propanol (mg/L) − 0.0407862 * 2-Phenyl ethanol (mg/L) + 0.168663 * Ethyl lactate (mg/L)	99.7145	0.0000
F4	Average age (years old) = 19.0455 − 0.00764656 * Potassium (mg/L) − 0.664414 * Total dry extract (g/L) + 1.15284 * Total reducing matter (g/L)	98.5339	0.0000
F5	Average age (years old) = 2.2474 − 0.000226935 * Glycerol (mg/L) + 0.00448994 * Acetaldehyde (mg/L) − 0.00146909 * Potassium (mg/L) + 1.86111 * Syringic acid (mg/L)	**99.9111**	**0.0000**

**Table 11:** Validation of the F5 model with five of the analyzed Fino samples.

Sample	Average Age (Years Old)	Forecast Age (Years Old)	Standard Forecast Error	Absolute Error (Years Old)
**6**	1	1.17622	0.0646336	−0.17622
**10**	2	2.19731	0.0581641	−0.19731
**15**	3	2.97550	0.0586390	0.02450
**19**	4	4.08016	0.0565806	−0.08016
**23**	5	5.04895	0.0607497	−0.04895

Samples 6, 10, 15, 19 and 23 are samples selected from the original data matrix to validate the proposed model and were not used in the MLR study.

**Table 12:** Regression models (MLR) to estimate the aging of Amontillado wines. A1: organic acids and glycerol model; A2: polyphenols model; A3: volatile compounds model; A4: “other variables” model; A5: overall model.

Model	Regression	R^2^ (Adjusted for DF)	*p*-Value Model (95%)
A1	Average age (years old) = −3.89092 − 0.0831495 * Citric acid (mg/L) + 0.19105 * Malic acid (mg/L) + 0.0328913 * Succinic acid (mg/L)	99.8702	0.0000
A2	Average age (years old) = 11.0696 + 4.07776 * Vanillic acid (mg/L) − 1.89923 * trans-coutaric acid + 1.45669 * Syringaldehyde (mg/L)	99.9456	0.0000
A3	Average age (years old) = −48.6603 + 0.029585 * Acetaldehyde (mg/L) − 0.0506701 * Ethyl lactate (mg/L) + 0.975076 * Diethyl succinate (mg/L) + 4.13569 * Ethyl myristate (mg/L) + 6.14993 * Ethyl palmitate (mg/L)	99.9050	0.0000
A4	Average age (years old) = −12.3184 − 4.80344 * Sulfate(g K_2_SO_4_/L) + 0.0122141 * Potassium (mg/L) + 0.398934 * Total dry extract(g/L) + 27.9698 * Absorbance 470 nm (a.u.)	99.8177	0.0000
**A5**	Average age (years old) = −19.7424 + 2.07137 * Vanillic acid (mg/L) + 0.399041 * Diethyl succinate (mg/L) − 3.37782 * Sulfate (g K_2_SO_4_/L) + 0.00783484 * Potassium (mg/L)	**99.9489**	**0.0000**

**Table 13:** Validation of the A5 model with five of the analyzed Amontillado samples.

Sample	Average Age (Years Old)	Forecast Age (Years Old)	Standard Forecast Error	Absolute Error (Years Old)
**26**	8	7.7019	0.3062	0.2981
**31**	12	12.0571	0.2682	−0.0571
**36**	16	15.9034	0.2685	0.0966
**41**	30	30.0863	0.3471	−0.0863
**46**	40	40.8162	0.2935	−0.8162

Samples 26, 31, 36, 41 and 46 are samples selected from the original data matrix to validate the proposed model and were not used in the MLR study.

**Table 14:** Tasting panel scores for the different descriptors of the four wines evaluated. Values are expressed as mean ± standard deviation. The *p*-values resulting from the application of 1-factor analysis of variance, taking the sample as the factor of variation, are included. Different superscripts in the scores indicate significant differences among the corresponding wines. The loadings of the descriptors after factor analysis (correlation coefficients with the extracted factors) are also shown.

Wine	Aromatic Intensity	Yeast	Nuts	Oak	Dryness	Equilibrium	Persistence
Young fortified wine (vintage)	2.4 ± 0.5 ^a^	1.6 ± 0.5 ^a^	1.0 ± 0.0 ^a^	1.0 ± 0.0 ^a^	2.0 ± 0.6 ^a^	2.1 ± 0.4 ^a^	2.0 ± 0.6 ^a^
Fino Sherry 5 years old	6.7 ± 0.5 ^b^	7.9 ± 0.7 ^c^	7.6 ± 0.5 ^c^	2.3 ± 0.5 ^b^	4.9 ± 0.7 ^b^	7.1 ± 0.7 ^b^	5.3 ± 0.5 ^b^
Amontillado Sherry 12 years old	5.9 ± 0.7 ^b^	3.6 ± 0.5 ^b^	6.4 ± 0.5 ^b^	6.0 ± 0.8 ^c^	7.7 ± 0.5 ^c^	6.4 ± 0.5 ^b^	5.9 ± 0.7 ^b^
Amontillado Sherry >30 years old	8.6 ± 0.5 ^c^	1.4 ± 0.5 ^a^	8.6 ± 0.5 ^d^	7.4 ± 0.8 ^d^	7.0 ± 0.8 ^c^	8.6 ± 0.5 ^c^	8.7 ± 0.5 ^c^
p_ANOVA_	0.000	0.000	0.000	0.000	0.000	0.000	0.000
Factor 1 loadings	0.942	0.063	0.938	0.887	0.893	0.949	0.967
Factor 2 loadings	0.174	0.983	0.304	−0.405	−0.084	0.238	−0.068

Mean values ± standard deviation (n = 7) are shown. For a given parameter, ANOVA: different letters (a, b, c, d) indicate significant differences (*p* < 0.05).

## Data Availability

Not applicable.

## Sample Availability

Samples of the compounds are not available from the authors.

## References

[1] López-Toledano A., Mayen M., Mérida J., Medina M. (2001). Compuestos fenolicos y color en tres tipos de vinos generosos sometidos a diferentes procesos de envejecimiento. Inf. Tecnol..

[2] Zea L., Moyano L., Moreno J., Cortes B., Medina M. (2001). Discrimination of the aroma fraction of Sherry wines obtained by oxidative and biological aging. Food Chem..

[3] Moyano L., Zea L., Moreno J.A., Medina M. (2010). Evaluation of the active odorants in Amontillado sherry wines during the aging process. J. Agric. Food Chem..

[4] European Parliament, Council of European Union (2019). Regulation (EC) 2019/787 of 17 April 2019, on the definition, description, presentation and labelling of spirit drinks, the use of the names of spirit drinks in the presentation and labelling of ther foodstuffs, the protection of geographical indications for spirit drinks, the use of ethyl alcohol and distillates of agricultural origin in alcoholic beverages, and repealing Regulation (EC) 110/2008. Off. J. Eur. Union.

[5] Aging. https://www.sherry.wine/sherry-wine/production/aging.

[6] Sherry Academy. https://www.sherry.wine/professionals/sherry-academy.

[7] Mangas J., Rodríguez R., Moreno J., Blanco D. (1996). Volatiles in Distillates of Cider Aged in American Oak Wood. J. Agric. Food Chem..

[8] Conner J.M., Paterson A., Piggott J.R. (1992). Analysis of lignin from oak casks used for the maturation of Scotch whisky. J. Sci. Food Agric..

[9] Pérez-Coello M.S., González-Viñas M.A., García-Romero E., Cabezudo M.D., Sanz J. (2000). Chemical and sensory changes in white wines fermented in the presence of oak chips. Int. J. Food Sci. Technol..

[10] Canas S. (2017). Phenolic Composition and Related Properties of Aged Wine Spirits: Influence of Barrel Characteristics. A Review. Beverages.

[11] Guerrero-Chanivet M., Valcárcel-Muñoz M.J., García-Moreno M.V., Guillén-Sánchez D.A. (2020). Characterization of the Aromatic and Phenolic Profile of Five Different Wood Chips Used for Aging Spirits and Wines. Foods.

[12] Cernîsev S. (2017). Analysis of lignin-derived phenolic compounds and their transformations in aged wine distillates. Food Control.

[13] Guillén D.A., Palma M., Natera R., Romero R., Barroso C.G. (2005). Determination of the age of Sherry wines by regression techniques using routine parameters and phenolic and volatile compounds. J. Agric. Food Chem..

[14] Sánchez-Guillén M.M., Schwarz-Rodríguez M., Rodríguez-Dodero M.C., García-Moreno M.V., Guillén-Sánchez D.A., García-Barroso C. (2019). Discriminant ability of phenolic compounds and short chain organic acids profiles in the determination of quality parameters of Brandy de Jerez. Food Chem..

[15] García Moreno M.V., García Barroso C. (2002). Comparison of the evolution of low molecular weight phenolic compounds in typical Sherry wines: Fino, Amontillado, and Oloroso. J. Agric. Food Chem..

[16] International Organization of Vine and Wine (2011). Method OIV-MA-F1-03. Determination of the acquired alcoholic strength by volume (ASV) of concentrated musts (CM) and grape sugar (or rectified concentrated musts, RCM). Compendium of International Methods of Analysis.

[17] International Organization of Vine and Wine (2015). Method OIV-MA-AS313-01. Total Acidity. Compendium of International Methods of Analysis.

[18] International Organization of Vine and Wine (2015). Method OIV-MA-AS313-02. Volatile Acidity. Compendium of International Methods of Analysis.

[19] International Organization of Vine and Wine (2009). Method OIV-MA-AS312-05. Glycerol. Compendium of International Methods of Analysis.

[20] International Organization of Vine and Wine (2009). Method OIV-MA-AS323-04B. Sulfur dioxide. Compendium of International Methods of Analysis.

[21] International Organization of Vine and Wine (2009). Method OIV-MA-AS321-05A. Sulfates. Compendium of International Methods of Analysis.

[22] International Organization of Vine and Wine (2009). Method OIV-MA-AS321-04. Total Phosphorus. Compendium of International Methods of Analysis.

[23] International Organization of Vine and Wine (2009). Method OIV-MA-AS2-03A. Total Dry Matter. Compendium of International Methods of Analysis.

[24] International Organization of Vine and Wine (2009). Method OIV-MA-AS311-01A. Reducing substances. Compendium of International Methods of Analysis.

[25] Valcárcel-Muñoz M.J., Guerrero-Chanivet M., García-Moreno M.V., Rodríguez-Dodero M.C., Guillén-Sánchez D.A. (2021). Comparative Evaluation of Brandy de Jerez Aged in American Oak Barrels with Different Times of Use. Foods.

[26] International Organisation of Vine and Wine (2009). Method OIV-MA-AS2-10. Folin-Ciocalteau Index. Compendium of International Methods of Analysis.

[27] Schwarz M., Rodríguez M.C., Guillén D.A., Barroso C.G. (2009). Development and validation of UPLC for the determination of phenolic compounds and furanic derivatives in Brandy de Jerez. J. Sep. Sci..

[28] International Organization of Vine and Wine (2009). Method OIV-MA-AS2-07B. Chromatic Characteristics. Compendium of International Methods of Analysis.

[29] (2007). Sensory Analysis—General Guidance for the Design of Test Rooms.

[30] (1977). Sensory Analysis—Apparatus—Wine-Tasting Glass.

[31] (2006). Sensory Analysis—Guidelines for the Use of Quantitative Response Scales.

[32] Zara G., Angelozzi D., Belviso S., Bardi L., Goffrini P., Lodi T., Budroni M., Mannazzu I. (2009). Oxygen is required to restore flor strain viability and lipid biosynthesis under fermentative conditions. FEMS Yeast Res..

[33] Carrascal García V. (2004). Estudio de los Ácidos Orgánicos en Brandy de Jerez y su Relación con las Prácticas Tradicionales de Elaboración.

[34] Swiegers J.H., Bartowsky E.J., Henschke P.A., Pretorius I.S. (2005). Yeast and bacterial modulation of wine aroma and flavour. Aust. J. Grape Wine Res..

[35] Moreno-Arribas M.V., Carmen-Polo M. (2008). Occurrence of lactic acid bacteria and biogenic amines in biologically aged wines. Food Microbiol..

[36] Roldán A., Lasanta C., Caro I., Palacios V. (2012). Effect of lysozyme on “flor” velum yeasts in the biological aging of sherry wines. Food Microbiol..

[37] Cordero-Bueso G., Ruiz-Muñoz M., González-Moreno M., Chirino S., Bernal-Grande M.C., Cantoral J.M. (2018). The microbial diversity of Sherry wines. Fermentation.

[38] Lasanta C., Gómez J. (2012). Tartrate stabilization of wines. Trends Food Sci. Technol..

[39] International Organization of Vine and Wine (2015). Treatment with calcium sulphate (plastering) (3/85). International Code of Oenological Practices.

[40] García-Ruiz J.M., Alcántara R., Martín J. (1995). Effects of Reutilized Potassium Bitartrate Seeds on the Stabilization of Dry Sherry Wine. Am. J. Enol. Vitic..

[41] Martínez E., Caro I., Bonat M., Pérez L., Domecq B. (1987). Dry extract in sherry and its evolution in the aging process. Am. J. Enol. Vitic..

[42] Le Floch A., Jourdes M., Teissedre P.-L. (2015). Polysaccharides and lignin from oak wood used in cooperage: Composition, interest, assays: A review. Carbohydr. Res..

[43] Sarni F., Moutounet M., Puech J.L., Rabier P. (1990). Effect of heat treatment of oak wood extractable compounds. Holzforschung.

[44] Martínez-Montero C., Rodríguez-Dodero M.C., Guillén-Sánchez D.A., García-Barroso C. (2005). Sugar contents of Brandy de Jerez during its aging. J. Agric. Food Chem..

[45] Alexandre H. (2013). Flor yeasts of Saccharomyces cerevisiae—Their ecology, genetics and metabolism. Int. J. Food Microbiol..

[46] Moreno J.A., Zea L., Moyano L., Medina M. (2005). Aroma compounds as markers of the changes in sherry wines subjected to biological aging. Food Control.

[47] Martínez P., Valcárcel M.J., Pérez L., Benítez T. (1998). Metabolism of Saccharomyces cerevisiae flor yeasts during fermentation and biological aging of Fino sherry: By-products and aroma compounds. Am. J. Enol. Vitic..

[48] Valcárcel-Muñoz M.J. (2020). Estudio de la Composición del “Velo de Flor” en un Soleraje de Fino.

[49] Zara S., Gross M.K., Zara G., Budroni M., Bakalinsky A.T. (2010). Ethanol-independent biofilm formation by a flor wine yeast strain of saccharomyces cerevisiae. Appl. Environ. Microbiol..

[50] Fabios M., Lopez-Toledano A., Mayen M., Merida J., Medina M. (2000). Phenolic compounds and browning in sherry wines subjected to oxidative and biological aging. J. Agric. Food Chem..

[51] Merida J., Lopez-Toledano A., Marquez T., Millan C., Ortega J.M., Medina M. (2005). Retention of browning compounds by yeasts involved in the winemaking of sherry type wines. Biotechnol. Lett..

